# Reduction of Salt and Sugar Contents in Canteen Foods and Intakes By Students and Staff at a Malaysian Higher Education Institution: Protocol for a Mixed Methods Study

**DOI:** 10.2196/69610

**Published:** 2025-07-21

**Authors:** Yook Chin Chia, Yee-How Say, Maong Hui Cheng, Felicia Fei Lei Chung, Tze Pheng Lau, Pei Boon Ooi

**Affiliations:** 1 Department of Clinical Medicine and Surgery, Sir Jeffrey Cheah Sunway Medical School Faculty of Medical and Life Sciences Sunway University Bandar Sunway Malaysia; 2 Ageing, Health and Well-Being Research Centre Faculty of Medical and Life Sciences Sunway University Bandar Sunway Malaysia; 3 Department of Primary Care Medicine Faculty of Medicine University of Malaya Kuala Lumpur Malaysia; 4 Department of Biomedical Sciences, Sir Jeffrey Cheah Sunway Medical School Faculty of Medical and Life Sciences Sunway University Bandar Sunway Malaysia; 5 School of Psychology Faculty of Medical and Life Sciences Sunway University Bandar Sunway Malaysia

**Keywords:** Nutritional intervention, workplace intervention, salt reduction, sugar reduction, study design

## Abstract

**Background:**

University students and staff members, especially those who frequently eat out and spend considerable time on campus, form a crucial demographic facing challenges related to high salt and sugar intake in out-of-home food. Recognizing the high prevalence of eating out among these populations, it is imperative to understand the salt and sugar consumption levels of campus communities.

**Objective:**

This protocol describes the rationale and design of a 3-part cross-sectional and longitudinal interventional study to reduce salt and sugar contents in canteen foods and intakes among students and staff at Sunway University and Sunway College in Malaysia.

**Methods:**

First, the knowledge, attitudes, and practices (KAPs) and perception, barriers, and enablers (PBEs) of salt and sugar intake and reduction were assessed among students and staff (part 1 of the study). Second, the KAPs and PBEs of salt, oil, and sugar reduction were assessed among canteen staff (part 2 of the study). Third, a longitudinal interventional study was conducted by implementing a campus-wide executive order to reduce salt and sugar in all foods sold on campus (part 3 of the study). The salt and sugar contents of selected foods were measured at baseline and at 3 months and 6 months postreduction. Participants who eat frequently on campus were selected as the intervention group, while those who do not comprised the control group. All participants had their urine electrolytes and body compositions measured and recorded 24-hour dietary intakes for 2 weekdays and 1 weekend at baseline, 3 months, and 6 months after salt and sugar reduction.

**Results:**

The study protocols were approved by the institutional review board of Sunway University (SUREC 2024/029 and SUREC 2024/040). Recruitment for the cross-sectional studies began in May 2024, while that for the longitudinal intervention study began in June 2024. The 6-month intervention began in September 2024 immediately after the official launch of the campus-wide executive order to reduce salt and sugar. We targeted recruitment of 1000, 50, and 300 participants for parts 1, 2, and 3, respectively. We anticipate reduced dietary salt and sugar intakes by 30% and 50%, respectively (World Health Organization targets), and beneficial health effects on the participants.

**Conclusions:**

The insights gained from this study will help to create a healthier food environment, benefitting individuals who regularly eat out, especially at the workplace.

**Trial Registration:**

ClinicalTrials.gov NCT06473038; https://clinicaltrials.gov/study/NCT06473038

**International Registered Report Identifier (IRRID):**

DERR1-10.2196/69610

## Introduction

### Background

Cardiovascular disease stands as a significant global health challenge, ranking among the primary causes of premature death and disability, especially in low-income countries. The burden of cardiovascular disease persists as the primary cause of death globally, including in Malaysia [1]. In Malaysia, hypertension has consistently emerged as a leading contributor to premature mortality. The 2023 National Health and Morbidity Survey (NHMS) revealed a 29.2% prevalence of hypertension among Malaysian adults aged 18 years and older [1]. Alarmingly, although the prevalence of hypertension is associated with older age, 80% of hypertensive adults aged between 18-29 years were unaware they had the condition [1].

A direct and positive association between salt intake and blood pressure (BP) regulation has long been well-established. From the initial International Study of Salt, Potassium, and Blood Pressure involving over 10,000 participants [2] to the largest United Kingdom Biobank study involving more than 450,000 adult participants [3], a significant association between 24-hour urinary sodium excretion (as a gold standard for estimating salt intake) and BP at the individual level has been established. A systolic BP increase of 2 to 3 mmHg for each 1 g/day increment in estimated salt excretion was generally reported [3].

According to the Malaysian Community Salt Survey study, the mean intake of salt in Malaysia is 7.9 g/day [4], much higher than the recommendation by the World Health Organization (WHO) of less than 5 g/day [5]. In fact, the WHO has is targeting a reduction in population salt intake of 30% by 2025 [6]. According to the same Malaysian survey, 64.4% of participants consumed at least 1 meal out-of-home, while 20.8% of participants reported consuming all 3 meals (breakfast, lunch, and dinner) out-of-home [7]. Pertaining to the occasion of a meal consumed out-of-home, slightly more than half (55% of respondents) consumed breakfast out-of-home, followed by lunch (35.6%) and dinner (27.1%) [8]. In 2019, household spending on out-of-home foods increased to 11.2% from 8.7% in 2004 and 2005 [9].

Besides hypertension, the prevalence of other noncommunicable diseases in Malaysia is also on the rise. According to the 2023 NHMS, 15.6% of the population has diabetes, and 54.4% are either overweight or obese [1]. The 2019 NHMS revealed that over one third of Malaysian adults consume commercial, ready-to-drink beverages at least once a week [10]. This unhealthy habit is even more prevalent among adolescents and young adults. On average, these groups consume 56.9 g of sugar daily, equivalent to about 12 teaspoons, primarily from various sugar-sweetened beverages (SSBs) [10]. Almost half of this sugar intake comes from commercially packaged, ready-to-drink beverages like carbonated soft drinks (“sodas”) and sweetened Asian drinks [10]. The WHO advises that adults and children should limit their daily intake of free sugars to less than 10% of their total energy intake [11]. For an average adult, this equates to no more than 50 g, or 12 teaspoons, of free sugar per day. Further reducing free sugar intake to below 5%, or around 25 grams (6 teaspoons), per day can offer additional health benefits [11]. When considering free sugars from other sources, such as processed and homemade foods and beverages, the total free sugar intake of Malaysians who regularly consume SSBs likely exceeds WHO recommendations. Malaysia has implemented the sugar tax of 40 sen (US $0.09) per liter in July 2019, which applies to two main categories of SSBs: beverages containing more than 5 g of sugar per 100 mL and fruit- or vegetable-based drinks with over 12 g of sugar per 100 mL [12]. In 2024, the sugar tax increased by 25% or 10 sen (US $0.02) to 50 sen (US $0.11) per liter [13].

From a study conducted among Malaysian consumers and food operators, the high salt in foods consumed out-of-home was acknowledged and a comprehensive salt reduction policy involving all stakeholders is needed [14]. Consumers face limited awareness and knowledge, counterproductive practices among food operators, and challenges in accessing affordable low-salt products, while food operators encounter a lack of standardized guidelines, ineffective enforcement, and uncooperative consumer practices [14].

University students and staff members, especially those who frequently eat out and spend considerable time on campus, form a crucial demographic facing challenges related to high salt and sugar intake in out-of-home food. Recognizing the high prevalence of eating out among these populations, it is imperative to understand the salt and sugar consumption of campus communities. Furthermore, serving low-salt foods at workplaces has been shown to reduce salt intake by 1.4 g/day [15].

Therefore, this study aims to assess the effects of salt and sugar reduction in foods sold on campus at Sunway University and Sunway College, higher education institutions located near Kuala Lumpur, the capital of Malaysia. These workplaces and academic campuses serve as an out-of-home food environment where the campus community heavily depends on vendors for their meals. The insights gained will not only benefit the individuals who regularly eat out-of-home, but will also contribute to creating a healthier food landscape on university campuses, reducing BP and impacting the overall well-being of the individuals.

### Specific Objectives of the Study

The study is divided into 3 parts, each with its own specific objectives.

#### Part 1

Part 1 of the study will explore the knowledge, attitudes, and practices (KAPs) and perception, barriers and enablers (PBEs) of dietary salt and sugar intake among Sunway University and Sunway College community (students and staff). This part of the study has four objectives, which are as follows:

1. To assess participants’ KAPs regarding salt consumption.
2. To assess participants’ KAPs regarding sugar-sweetened beverage consumption and attitudes toward sugar taxation in Malaysia.
3. To assess participants’ KAPs regarding diabetes mellitus.
4. To identify common PBEs in implementing salt and sugar reduction on campus.

#### Part 2

Part 2 of the study will explore the KAPs and PBEs of salt, oil, and sugar (SOS) reduction among campus canteen staff. This part of the study has two objectives, which are as follows:

1. To investigate campus canteen staff’s KAPs regarding SOS usage and intake.
2. To identify PBEs in implementing SOS reduction in the foods sold by campus canteen staff.

#### Part 3

Part 3 is an interventional study on the reduction of salt and sugar in foods sold on campus. This part of the study will be divided into studies A and B, as described below.

#### Study A

Study A will investigate the salt and sugar contents of selected food sold on campus. This part of the study has three objectives, which are as follows:

1. To survey and compile a comprehensive list of existing foods sold at Sunway University and Sunway College.
2. To analyze the salt and sugar contents of the selected foods at baseline, 3 months, and 6 months after the campus-wide executive order to reduce salt and sugar is implemented.
3. To propose further recommendations for reducing salt and sugar content in foods sold on campus.

#### Study B

Study B will investigate the effects of salt and sugar reduction on the participants. This part of the study has three objectives, which are as follows:

1. To investigate dietary salt intake using dietary records and spot urine tests in a subset of participants at baseline and after a campus-wide executive order to reduce salt and sugar in all foods sold on campus is implemented.
2. To investigate the perceived intensity and pleasantness of saltiness of the participants in the intervention and control groups at baseline and after the campus-wide executive order is implemented.
3. To investigate the effect on the participants’ anthropometric measurements and body composition at baseline and after the campus-wide executive order is implemented.

## Methods

### Study Protocol

**Figure 1:**
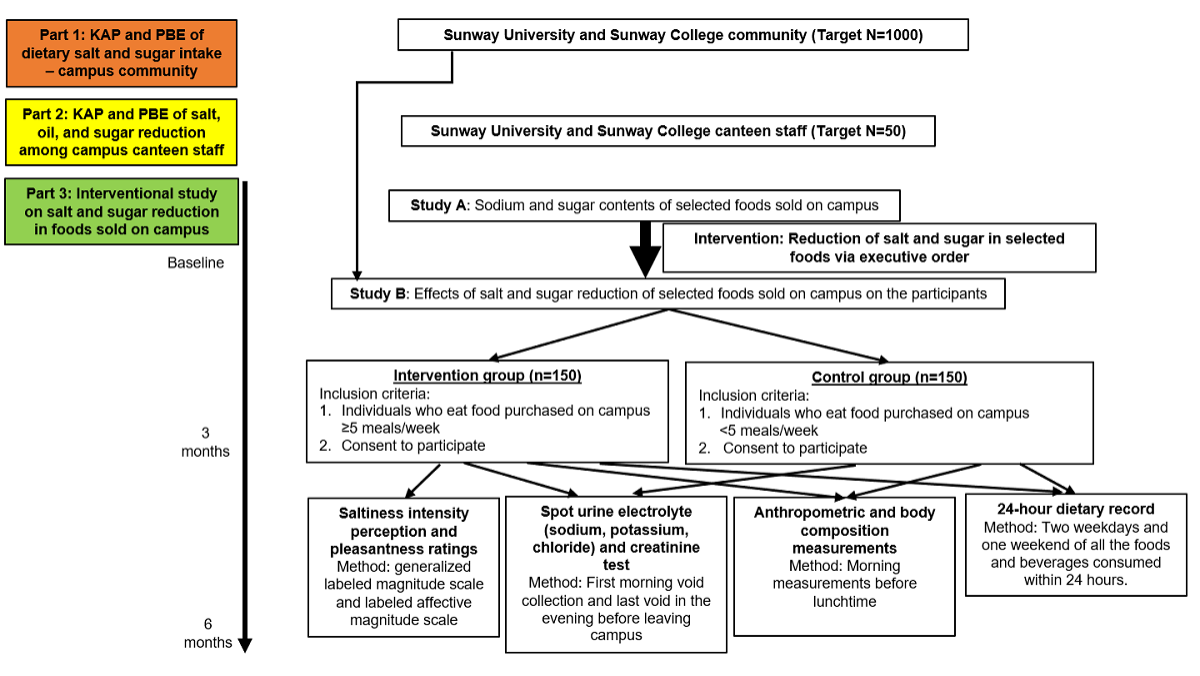
Flow chart of the study design. KAP: knowledge, attitudes, and practices; PBE: perception, barriers, and enablers.

The SPIRIT (Standard Protocol Items: Recommendations for Interventional Trials) checklist is found in Multimedia Appendix 1.The study protocol described herein was approved by the institutional review board of Sunway University, with the approval codes SUREC 2024/029 and SUREC 2024/040. The approved protocols are found in Multimedia Appendices 2 and 3, respectively. The study is funded by the Malaysia Society of Hypertension (MSH 2024/01), who has peer-reviewed the proposal and granted approval on the amended proposal (Multimedia Appendix 4). The study has been registered at ClinicalTrials.gov, with the identifier number NCT06473038. This 3-part nutritional intervention study titled “An Interventional Study of Salt and Sugar Reduction in Foods Sold on Campus at Sunway University and Sunway College” was carried out from May 2024 to April 2025. First, a cross-sectional study on the KAPs and PBEs of salt and sugar intake among Sunway University and Sunway College students and staff was assessed. Second, a cross-sectional study on the KAPs and PBEs of salt, oil, and sugar (SOS) reduction among campus canteen staff was conducted. Third, a prospective, longitudinal study involving an interventional reduction of salt and sugar in all foods sold on campus was conducted by assessing the salt and sugar contents of selected foods sold on campus before and after imposing a campus-wide executive order (the intervention) to reduce the salt and sugar in all foods. Those who eat frequently on campus (intervention group) and those who do not (control group) were recruited to participate in the interventional study. A flow chart of the study design is shown in Figure 1. This study only involved students and staff at Sunway University and Sunway College, Sunway City, Selangor, Malaysia.

### Ethical Considerations

Informed consent with identifying information omitted (except for email addresses for follow-up purposes) was obtained from all participants. The study was conducted according to the Declaration of Helsinki; participants’ data were handled securely, and confidentiality was maintained. The study protocols were approved by the institutional review board of Sunway University (SUREC 2024/029 and SUREC 2024/040).

Compensation was provided as follows: for part 1 of the study, each participant was given a voucher worth MYR 10 (US $2.20) and the choice to enter a lucky draw to win 3 lucky draw food hampers worth MYR 200 (US $43.00) each; for part 2, each participant was given a voucher worth MYR 10 (US $2.20) upon successful completion of the questionnaire; for part 3, each participant was given MYR 10 (US $2.20) per time point (ie, at the start of the study, after 3 months, and after 6 months) for successful completion of the questionnaire, dietary records, anthropometric and body composition measurements, saltiness intensity perception and pleasantness ratings, and for providing urine samples (totaling MYR 30 or US $6.40 per participant).

### Study Procedures

#### Part 1: KAPs of Dietary Salt and Sugar Intake

##### Sample Size Calculation

Using the Raosoft sample size web-based calculator [16], we determined that a minimum sample size of 370 was needed in order to achieve a 5% margin of error and 95% CI given a Sunway University and Sunway College population size of around 10,000 and a 50% response rate. Therefore, a target sample size of 1000 participants was set to account for midstudy dropouts. Recruitment took place from May to June 2024.

##### Instrument

Participants were invited to complete a web-based Google Form questionnaire (Multimedia Appendix 5) to assess the following:

1. Sociodemographic and lifestyle factors such as age, gender, ethnicity, education level, medical history, and smoking and alcohol-drinking practices
2. The frequency of eating meals on campus and general perceptions of the saltiness and sweetness levels of foods sold on campus
3. KAPs toward dietary salt (adopted from [17])
4. KAPs toward SSBs (adopted from [18]) and sugar tax attitudes (adopted from [19])
5. PBEs for implementing salt reduction (adapted from [14])
6. Knowledge of diabetes mellitus using a patient diabetes knowledge questionnaire (adopted from [20])
7. Diabetes Risk Score, a simple, fast, inexpensive, noninvasive, and reliable tool to identify individuals at high risk for type 2 diabetes (adopted from [21]).

A booth was set up in the campus lobby with high foot traffic. A promotional banner with a QR code that linked to the Google Form was set up, and promotional flyers were distributed to passersby. Promotion was also conducted through web-based course announcement boards and word-of-mouth, especially among academic staff.

##### Inclusion Criteria

Part 1 of the study included students and staff of Sunway University and Sunway College aged 18 years and older who understand English and were able to provide written informed consent via a Google Form.

##### Exclusion Criteria

Individuals with an acute illness or psychological or psychiatric condition that may affect their ability to answer the survey questions objectively were excluded.

#### Part 2: KAPs and PBEs of SOS Reduction by Campus Canteen Staff

##### Sample Size Calculation

The estimated number of campus canteen staff that are involved in food preparation was around 65 people, and given an estimated response rate of 80%, the targeted number of participants was approximately 50 people. A townhall meeting with all the food vendors was held at a lecture hall, where the principal investigators explained the purpose of the study and conducted the pen-and-paper questionnaire on the spot. Recruitment took place from May to June 2024.

##### Instrument

A questionnaire on KAPs on SOS usage in food preparation (including on campus) and in habitual dietary intake by campus canteen staff was adopted from [22], while a questionnaire on PBEs to salt and sugar reduction by campus canteen staff was adopted from [14] (Multimedia Appendix 6).

##### Inclusion Criteria

Part 2 of the study included canteen staff aged 18 years and older who were involved in food preparation and able to provide written informed consent via a Google Form.

##### Exclusion Criteria

Individuals with an acute illness or psychological or psychiatric condition that may affect their ability to answer the survey questions objectively were excluded.

#### Part 3: Interventional Study on Salt and Sugar Reduction in Foods Sold on Campus.

##### Study A

Selected samples of food sold on campus were analyzed for their salt and sugar contents at baseline and at 3 months and 6 months after the campus-wide executive order to reduce salt and sugar in foods was implemented. Baseline collection took place from May to June 2024. The executive order was implemented in September 2024, and sample collection took place in December 2024 (3 months after the executive order) and March 2025 (6 months after the executive order). Compliance with the executive order was monitored solely based on the salt and sugar contents of the foods 3 months and 6 months after implementation of the executive order.

###### Food Sample Collection and Measurement Procedures

A targeted number of 4-5 food samples with the most popular sales numbers (verbally confirmed with vendors) per vendor for a total of 17 vendors on the campus were collected in resealable Ziplock storage bags. Salt levels in foods were measured by inductively coupled plasma-optical emission spectroscopy using the service of an outsourced International Organization for Standardization (ISO)/International Electrotechnical Commission (IEC) 1702-certified commercial laboratory (ALS Technichem, Malaysia). A complementary method of salt measurement in foods will be performed using the PAL-SALT pocket salt meter (Atago, Japan). The sugar profile of foods included the amounts of simple sugars (ie, fructose, galactose, glucose, sucrose, maltose, and lactose). The sugars were extracted and purified and the appropriate dilution was quantitated using high-performance liquid chromatography using the service of an outsourced ISO/IEC 1702-certified commercial laboratory (Permulab, Malaysia).

##### Study B

Before the actual lowering of salt and sugar in foods sold on campus, all the food vendors were briefed on the amount of salt and sugar to be reduced for all the foods sold.

###### Sample Size Calculation

According to the WHO/Pan American Health Organization Regional Expert Group for Cardiovascular Disease Prevention through Population-wide Dietary Salt Reduction Subgroup for Research and Surveillance [17], to meet the 0.5 g difference in sodium (salt), a sample size of 150 participants per arm was required. Therefore, a total of 150 participants who often ate foods sold on campus, defined as those who ate at least 5 meals of campus foods per week, were selected for part 1 of the study in a consecutive manner; they formed the intervention group who ate foods sold on campus that had reduced salt and sugar. Another 150 participants who did not often eat foods sold on campus, defined as those who ate less than 5 meals of campus foods per week, made up the control group.

Participants had to provide urine samples for the measurement of urinary salt, potassium, chloride, and creatinine; have body composition and anthropometric measurements taken, keep 24-hour dietary records, and complete saltiness intensity perception and pleasantness ratings of the foods on campus at the start of the study (baseline) and at 3 months and 6 months after the executive order (Multimedia Appendix 7). Recruitment and baseline measurements took place from June to August 2024, while measurements at 3 months and 6 months after the executive order were collected in December 2024 and March 2025, respectively. Data monitoring, harm monitoring, and trial auditing were required as this study did not involve any administration of a drug or therapy in a clinical setting.

###### Inclusion Criteria for the Intervention Group

Individuals who provided informed consent and ate at least 5 meals of campus food per week were included in the intervention group. This frequency was based on the average number of meals (including breakfast, lunch, dinner, and snacks) per day eaten on campus, which was determined in a pilot study of 100 people. The majority of the participants who ate on campus only did so on weekdays and did not have their dinners there.

###### Inclusion Criteria for the Control Group

Individuals who provided informed consent and did not eat campus food or ate campus food for <5 meals per week were included in the control group.

###### Exclusion Criteria

Individuals with an acute illness or psychological or psychiatric conditions were excluded.

###### 24-Hour Dietary Records

Participants were instructed to keep 24-hour dietary records for 2 weekdays and 1 weekend of the same week by writing down all foods and beverages consumed during a 24-hour period (each day starting at 12:00 AM and ending at 11:59 PM). Participants were asked to list the approximate time the meal was consumed, the place where it was consumed (eg, at home, on campus, or at a specific restaurant), and the type of eating occasion or meal (breakfast, lunch, dinner, snack, or other). The list of each food or beverage consumed included foods eaten between meals and all drinks, even noncaloric items like water. Specific details, ingredients, preparation methods, and brand names of each food or beverage consumed were also listed. Participants were instructed to pay attention to any sauce or gravy in the food, as these are usually high in salt and sugar. The portion size of each food or beverage item consumed was recorded using an appended “Food Portion Size” guide.

#### Urine Analysis

The intervention and control participants provided 2 spot urine samples—the first morning void and the last void in the late afternoon or early evening prior to the evening meal—at each of the 3 time points (baseline, after 3 months, and after 6 months). Urine electrolytes (salt, potassium, and chloride) and creatinine were measured using the potentiometry method (Alinity ci-series, Abbott) at the Laboratory Department of Sunway Medical Centre, Bandar Sunway. The average of the morning and afternoon readings was used to estimate 24-hour urine salt levels, using a previously published formula validated for the Malaysian population [23].

#### Assessment of Saltiness Intensity Perception and Pleasantness Ratings

Participants received verbal and written English instructions and then completed 2 rating scales assessing their perceived intensity of saltiness of the selected foods sold on campus (hereafter referred to as “intensity perception”) and the extent to which they liked or disliked this level of saltiness (hereafter referred to as the “pleasantness rating”). The perceived saltiness intensity was measured by using the generalized labeled magnitude scale [24]; participants were asked to make a horizontal marking on a paper version of the scale. A 100-mm scale was constructed with 6 descriptors ranging from “barely detectable” to “strongest imaginable sensation of any kind.” For pleasantness, the labeled affective magnitude scale [25] was used. It is a 100-mm scale that is constructed with 11 different levels ranging from “greatest imaginable disliking” to “greatest imaginable liking.”

#### Anthropometric and Body Composition Measurements

Clinical measurements indicative of vascular health, namely, systolic blood pressure, diastolic blood pressure, and pulse rate, were taken using an automated blood pressure monitor (HEM-7121, Omron, Japan) after the participants had rested for 5 minutes. The height, waist circumference, and hip circumference of each participant were measured using a measuring tape, and the waist-hip ratio was calculated by dividing waist circumference by hip circumference. A bioimpedance body composition scale (HBF-375 Karada Scan, Omron, Japan) was used to determine weight, body mass index (BMI), total body fat, visceral fat, subcutaneous fat, skeletal muscle percentage, and resting metabolism rate. Based on previously published data for the Asian population, participants ≥23 kg/m^2^ will be considered to be overweight and those ≥25 kg/m^2^ will be considered to be obese [26]. The following previously published cutoff points relevant to the Asian population will also be used to summarize the results: for overall adiposity (total body fat) the cutoffs will be 20% for male participants or 30% for female participants [27] and for central adiposity (waist-hip ratio) the cutoffs will be 0.90 for male participants or 0.85 for female participants [28].

#### Data Analysis

The data collected from the Google Forms will be captured on Google Sheets and analyzed using SPSS (version 27; IBM Corporation) and R (version 4.5.1; R Foundation for Statistical Computing) statistical software. The data will be cleaned, coded, and checked for missing values before analysis.

Continuous variables will be tested for normality using Shapiro-Wilk tests. Normally distributed continuous data will be summarized as means and SDs, while skewed data will be presented as medians and IQRs. Categorical variables will be reported as frequencies (percentages) and compared using chi-square tests or Fisher exact tests where appropriate.

To account for individual dietary variability, including meals consumed outside campus, several adjustments will be made. Linear mixed-effects models (LMMs) or generalized linear mixed models will be used to analyze changes in dietary salt and sugar intake, incorporating random effects for participants to account for repeated measures (ie, baseline, 3 months, and 6 months). The models will adjust for potential confounders, such as age, gender, BMI, baseline dietary intake, and frequency of eating on campus.

For primary outcomes of the intervention effect analysis, including changes in 24-hour dietary salt and sugar intake (from food diaries), changes in spot urine salt excretion (using validated estimation equations for the Malaysian population), and anthropometric measure and /body composition changes (eg, weight, BMI, and body fat percentage), the statistical tests that will be performed are LMMs to assess time-dependent changes in intake while adjusting for individual heterogeneity and generalized estimating equations for binary outcomes like “met recommended daily intake” (yes or no). For secondary outcomes, including saltiness intensity perception and pleasantness ratings and changes in blood pressure, we will use repeated measures ANOVA or LMMs to track sensory adaptation and paired *t* tests (if normal) or Wilcoxon signed-rank tests (if skewed). Subgroup analyses based on eating frequency, gender, and BMI categories will also be performed to minimize interaction effects.

All statistical tests will be 2-tailed, with significance set at *P*<.05. Adjustments for multiple comparisons will be made using Bonferroni correction where necessary.

## Results

**Figure 2:**
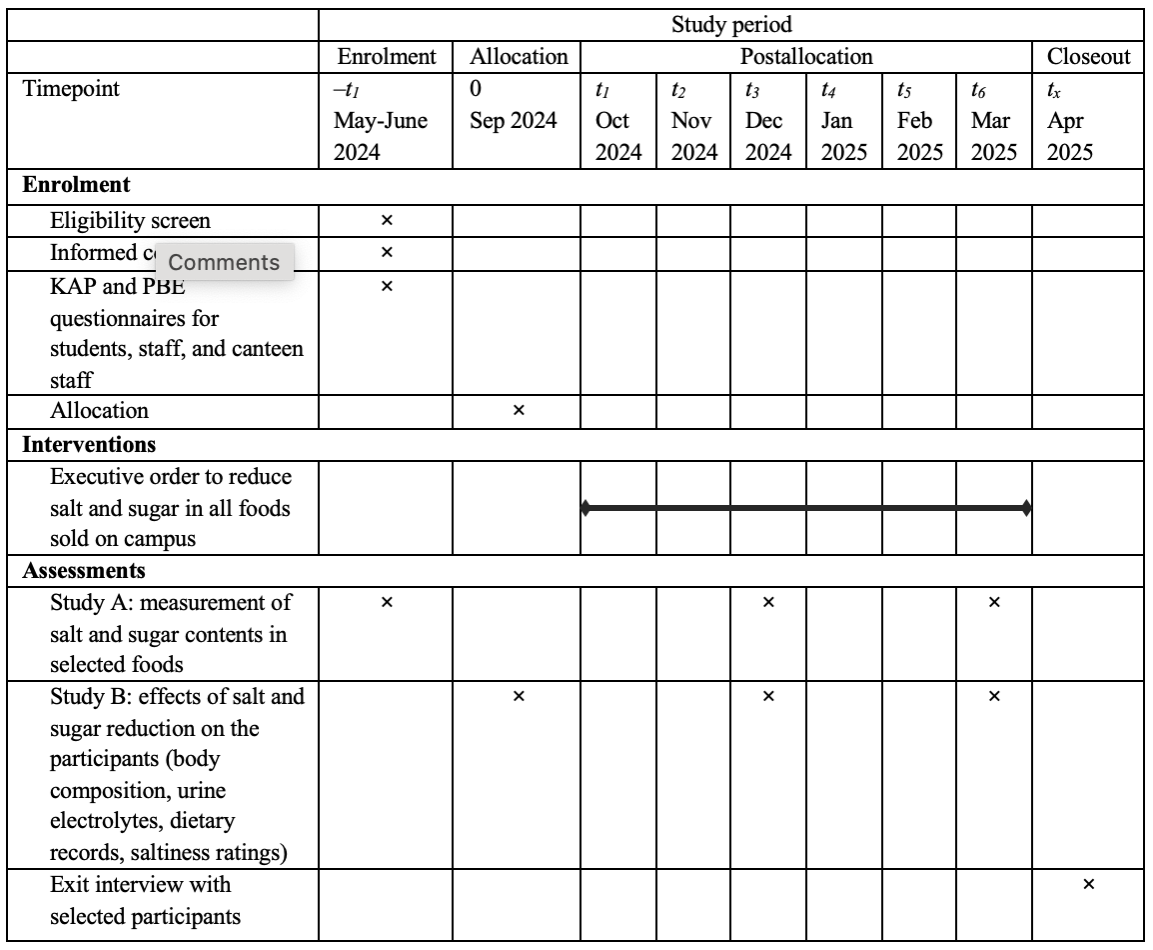
The schedule of enrolment, interventions, and assessments for the salt and sugar reduction study in foods sold on campus at Sunway University and Sunway College. KAP: knowledge, attitudes, and practices; PBE: perception, barriers, and enablers.

Recruitment for the cross-sectional studies (parts 1 and 2) began in May 2024. For the longitudinal intervention study (part 3), recruitment and baseline measurements took place from June to August 2024, and the 6-month intervention began in September 2024 immediately after the official launch of the campus-wide executive order. The timeline of the study is shown in Figure 2. There have been no deviations of the protocols since being registered in the ClinicalTrials.gov registry (NCT06473038) and published herein. There were variations on the actual sample sizes in all stages during the recruitment process, and these will be reported in our future publications on the findings of this study.

## Discussion

The food environment, which encompasses factors such as the presence, ease of access, price, quality, and advertising of specific food options, significantly influences what people consume [29]. An unhealthy food environment contributes to unhealthy eating patterns; therefore, altering the food environment holds promise for fostering and supporting healthy behaviors, serving as a foundation for workplace and academic campus health initiatives [30]. Workplaces and academic campuses are sedentary settings where energy-dense foods and beverages are commonly available [31]. Viewed from an economic perspective, there is increasing concern regarding the financial impact of cardiometabolic diseases within workplaces, stemming from expenses linked to absenteeism, sick leave, disability, injuries, and health care claims [32]. However, looking on the bright side, the workplace and academic campus offer strategic environments for implementing initiatives aimed at fostering healthy eating habits, considering that employees and students dedicate up to 60% of their waking hours there [30]. Therefore, the workplace and academic campus have the potential to reach a significant portion of adults, including those who may not typically participate in preventive health programs [33].

In a review of 55 interventional studies aimed to promote healthy eating at workplace cafeterias, it was found that these interventions resulted in a higher intake of fruits and vegetables, improved dietary intake, improved health outcomes, and improved healthy food sales [34]. Specifically, similar salt or salt reduction intervention studies that have been conducted among workplace employees or academic campus communities have yielded mainly positive results. For example, a study in Portugal found that there was no significant decrease in consumer satisfaction and in food waste generated [35]. Another study in Ireland found that lower percentages of participants exceeded the recommended salt intake (4-6 g/day) at the intervention hospital cafeteria than at the nonintervention hospital [36]. A study in Japan found that there was a reduction of 1.4 g in salt intake at the intervention workplace [15], whereas another study in Germany found a reduction of 1.2 g in salt intake among men (but not women) at the intervention workplace [37].

One of the strengths of this study is that, to the best of our knowledge, it will be the first study to administer salt and sugar interventions at an academic campus in Malaysia with a sizeable sample of apparently healthy and mostly young adults. Similar dietary intervention studies on salt reduction in Malaysia have so far been limited to middle-aged and older adults with elevated blood pressure [38] or conducted at a pilot scale among the staff of the Ministry of Health, Malaysia (My STARS study) [39]. Our research is innovative because Malaysia has not yet established an effective and comprehensive strategy to reduce salt intake in food consumed outside the home. Our methodology will facilitate data collection essential for formulating such a policy.

However, we do recognize a potential major limitation of the study. Spot urine collections will be adopted instead of a standard approach of 24-hour urine collection. We recognize that any single urine collection may not be representative of usual individual intake. However, this method is considered an acceptable proxy for intake at the group level as we are using it as a way to monitor change rather than to determine absolute levels [40]. Obtaining complete 24-hour urine collections every 3 months during a 6-month intervention is rare and remains challenging [41].

Overall, this project aims to foster a culture of health and well-being within the Sunway University and Sunway College community while contributing to broader public health objectives and corporate social responsibility. Through collaboration between students, staff, and the institution, the project seeks to drive positive change in dietary habits, ultimately promoting a healthier lifestyle. This will contribute toward WHO goals to reduce the intake of salt by 30% by 2025 and further the reduction of free sugar intake to below 5% of total energy intake (25 g or 6 teaspoons) per day and toward the United Nation’s third sustainable development goal focusing on good health and well-being.

## Appendix

Multimedia Appendix 1 SPIRIT (Standard Protocol Items: Recommendations for Interventional Trials) checklist.

Multimedia Appendix 2 Institutional review board of Sunway University ethical approval and approved protocol (SUREC 2024/029).

Multimedia Appendix 3 Institutional review board of Sunway University ethical approval and approved protocol (SUREC 2024/040).

Multimedia Appendix 4 Malaysia Society of Hypertension peer-reviewed report, grant approval, and proposal.

Multimedia Appendix 5 Knowledge, attitudes, and practices and perception, barriers, and enablers questionnaire for students and staff.

Multimedia Appendix 6 Knowledge, attitudes, and practices and perception, barriers, and enablers questionnaire for campus canteen staff.

Multimedia Appendix 7 Longitudinal intervention study form.

## Abbreviations

BP: blood pressure
BMI: body mass index
KAP: knowledge, attitudes, and practices
LMM: linear mixed-effects model
NHMS: National Health and Morbidity Survey
PBE: perception, barriers, and enablers
SOS: salt, oil, and sugar
SPIRIT: Standard Protocol Items: Recommendations for Interventional Trials
SSB: sugar-sweetened beverages
WHO: World Health Organization

## Data Availability

All relevant data from this study will be made available upon study completion.
