# Supplementary material for: Reduction of Salt and Sugar Contents in Canteen Foods and Intakes By Students and Staff at a Malaysian Higher Education Institution: Protocol for a Mixed Methods Study
Source: JMIR Res Protoc. 2025 Jul 21;14:e69610. doi: 10.2196/69610 (PMC12322606; doi:10.2196/69610)
Supplement: Multimedia Appendix 5 [file resprot_v14i1e69610_app5.docx]

**An Interventional Study of Salt and Sugar** **Reduction in Foods Sold On Campus at Sunway University and Sunway College**

We would like to invite you to take part in a research study. Before you decide whether to participate, you need to understand why the research is being done and what it would involve. Please take time to read the following information carefully; talk to others about the study if you wish.

Feel free contact the study team if there is anything that is not clear or if you would like more information. Take time to decide whether or not you wish to take part.

1. **What is the purpose of this study?**

This study aims to assess the effects of salt and sugar reduction in foods sold on campus at Sunway University and Sunway College. For the first part of study, the knowledge, attitude, and practice (KAP) of dietary salt and sugar intake among Sunway University and Sunway College students and staff will be assessed. For the second part of study, selected participants will be invited to participate in an interventional study for 6 months, where they will be exposed to foods sold on campus that have reduced salt and sugar.

1. **Why is this study important?**

University students and staff members on campus, especially those who frequently eat out and spend considerable time on campus, form a crucial demographic facing challenges related to high salt and sugar intake in out-of-home food. Recognizing the prevalence of this lifestyle among these populations, it becomes imperative to understand the perceptions and experiences of both students and campus communities regarding salt and sugar consumption. Furthermore, serving lowered salt foods at workplaces have been shown to be able to reduce salt intake by at least 0.5 gm/day.

In this study, we aim to shed light on the dynamics of the university campus food environment, where students and campus communities heavily depend on vendors for their meals. The insights gained will not only benefit the individuals who regularly eat out but will also contribute to creating a healthier food landscape on university campuses, impacting the overall well-being of the individuals. This study holds significance in addressing the shared responsibility between individuals who use out-of-home food and vendors in promoting a healthier lifestyle within the university community.

1. **What type of study is this?**

The first part of the study is a cross-sectional study on the KAP towards dietary salt and sugar intakes, and diabetes, and barriers and enablers for salt reduction.

The second part of the study is a 6-month interventional study, where selected participants will have different exposures towards the reduction of salt and sugar in foods sold on campus.

1. **What is the procedure that is being tested? (If applicable)**

This study does not involve the testing of any procedures/ products.

1. **Does the investigatory product contain culturally sensitive ingredients e.g.: bovine or porcine? (if applicable)**

This study does not involve the testing of any procedures/ products.

1. **Why have I been invited to participate in this study?**

We are inviting all individuals 18 years and above studying or working at Sunway University or Sunway College to participate.

1. **Who should not participate in the study?**

Individuals below 18 years old and who are unable to give informed consent should not participate in this study.

1. **Can I refuse to take part in the study?**

Yes, you are allowed to do so as this study is entirely voluntary.

1. **What will happen to me if I take part?**

The first part of the study is a cross-sectional study where you will be required to answer a series of questionnaires on socio-demographic and lifestyle factors, KAP towards dietary salt and sugar intakes, and diabetes, and barriers and enablers for salt reduction.

If you consent to and if selected to participate in the second part of the study, you will be required to provide urine samples for the measurement of urinary sodium, potassium, chloride, and creatinine, have your body composition and anthropometric measurements taken, perform 24-hour dietary records, perform saltiness intensity perception and pleasantness ratings of the food on campus, at three timepoints – at the start of the study, after 3 months, and after 6 months.

1. **How long will I be involved in this study?**

The first part of the study requires approximately 15 minutes.

The second part of the study requires a commitment of 6 months.

1. **What are the possible disadvantages and risks?**

Participants will not face any major risks in this study. Urine collection is non-invasive and does not pose any harm to the body.

1. **What are the possible benefits to me?**

The findings of the study could be used as ways to increase awareness of reducing salt and sugar intake at a workplace/studyplace.

1. **Who will have access to my medical records and research data?**

The researchers directly involved in this study will have access to the data collected in this study. Nevertheless, all measurements will be anonymized, and it will not be possible to link any of the data back to specific individuals.

1. **Will my records/data be kept confidential?**

All data collection will be anonymized, coded, and stored electronically in Sunway University in a password-protected le. The hard copy of the data will be kept in a locked cabinet in the Department of Medical Sciences.

1. **What will happen if I don’t want to carry on with the study?**

If you decide not to participate in the study, you may drop out voluntarily.

1. **What happens when the research study stops?**

The study team will analyze the data of the research study. No further interventions or contact will be made with the study participants.

1. **What will happen to the results of the research study?**

Data from the study will be analyzed, and the results of the study may be published in an academic journal and/or distributed through other media channels.

1. **Will I receive compensation for participating in this study?**

For the first part of the study, there will be no compensation but you are entitled to enter a lucky draw to win three lucky draw food hampers.

If you are invited to participate in the second part of the study, you will be compensated with RM 10 per person for successful completion of questionnaire, dietary records, anthropometric and body composition measurements, and providing urine samples at three timepoints - baseline, 3 months and 6 months (maximum RM 30 per participant).

1. **Who should I contact if I have additional questions/problems during the course of the study?**

Prof Dr Chia Yook Chin, Head of Department of Medical Sciences, School of Medical & Life Sciences, Sunway University. Email: ycchia@sunway.edu.my

* Indicates required question

1. Email *

# Inclusion Criteria & Consent Form

1. By joining this study, I confirm that I am (tick all that matters): *

*Check all that apply.*

A student or staff of Sunway University and Sunway College aged 18 years and above

Understands English

1. I understand the procedures described above. My questions have been answered to my * satisfaction, and I acknowledge that I am participating in this study of my own free will. I understand that I may refuse to participate or stop participating at any time.

By signing this consent form, I agree to the collection of my email address, solely for the purpose of lucky draw and for follow-up contact in Part 2 of the study. All my information obtained in this study will be kept and handled confidentially, in accordance with the Malaysian Personal Data Protection Act 2010 ("PDPA") Please tick all that matters.

*Check all that apply.*

Yes, I consent to participate in Part 1 of the study

Yes, I consent to participate in Part 2 of the study

# Part A: Socio-demographics, medical history & lifestyle factors

Please complete this part. Fill in the particulars or choose only one most relevant answer.

1. A1. Gender *

*Mark only one oval.*

Male

Female

1. A2. Age *
2. A3. ONE ethnicity that would best describe yourself as: *

*Mark only one oval.*

Malay

Chinese

Indian Other:

1. A4. Please select your **highest** educational level (do NOT consider the education level * you are currently undertaking).

*Mark only one oval.*

No formal education

Primary

Secondary

Tertiary - Pre-university/foundation/diploma

Tertiary - Undergraduate

Tertiary - Postgraduate

1. A5. Have you ever been diagnosed with high blood pressure or hypertension by a health * professional (except in pregnancy)?

*Mark only one oval.*

Yes

No

Don't know

1. A6. If yes to A5, at which age were you diagnosed?
2. A7. Have you ever been diagnosed as having diabetes by a health professional (except * in pregnancy)?

*Mark only one oval.*

Yes

No

Don't know

1. A8. If yes to A7, at which age were you diagnosed?
2. A9. Do you use tobacco? (including chewing tobacco, cigars and pipes) *

*Mark only one oval.*

Yes

No

Never

1. A10. Do you vape? *

*Mark only one oval.*

Yes

No

Never

1. A11. Do you drink alcohol? *

*Mark only one oval.*

Never

1 - 3 times per month

1 - 6 times per week

Daily

1. A12. Do you eat on campus? *

*Mark only one oval.*

Yes

No

1. A13. If Yes to A12, how many meals per week do you eat on campus for breakfast?

*Mark only one oval.*

1

2

3

4

5

>5

1. A14. If Yes to A12, how many meals per week do you eat on campus for lunch?

*Mark only one oval.*

1

2

3

4

5

>5

1. A15. If Yes to A12, how many meals per week do you eat on campus for dinner?

*Mark only one oval.*

1

2

3

4

5

>5

1. A16. Do you think the foods sold on campus are generally too salty? *

*Mark only one oval.*

Yes

Just right

No

Don’t know; I don’t consume foods sold on campus

1. A17. If Yes to A16, please provide some examples of stalls on campus that sell foods that are too salty.
2. A18. Do you think the beverages sold on campus are generally too sweet? *

*Mark only one oval.*

Yes

Just right

No

Don’t know; I don’t consume beverages sold on campus

1. A19. If Yes to A18, please provide some examples of beverages sold on campus that are too sweet.

# Part B: Questionnaire on KAP towards salt intake

The questions in this section aim to determine knowledge, attitudes and practices towards salt usage and intake. There are no right or wrong answers, and we value your answers with credibility and transparency.

1. B1. Do you add salt/tomato sauce/chili sauce/soy sauce/food enhancer to food at the * table?

*Mark only one oval.*

Never

Rarely

Sometimes

Often

Always

1. B2. In the food you eat at home, salt is added in cooking… *

*Mark only one oval.*

Never

Rarely

Sometimes

Often

Always

1. B3. How much salt do you think you consume? *

*Mark only one oval.*

Far too much

Too much

Just the right amount

Too little

Far too little

Don’t Know

Refused

1. B4. Do you think that a high salt diet could cause a serious health problem? *

*Mark only one oval.*

Yes

No

Don’t know

Refused

1. B5. If Yes to B4, what sort of problem? Do you think that a high salt diet could cause a serious health problem?

*Check all that apply.*

High blood pressure

Osteoporosis

Stomach cancer

Kidney stones

None of the above

All of the above

Don’t know

Refused

1. B6. How important to you is lowering the salt/sodium in your diet? *

*Mark only one oval.*

0 1 2 3 4 5 6 7 8 9 10

Very important

important

Not

1. B7. Do you do anything on a regular basis to control your salt or sodium intake?

*Mark only one oval.*

Yes

No

Don’t know

Refused

1. B8. If Yes to B7, what do you do? (You can answer more than one)

*Check all that apply.*

Avoid/minimize consumption of processed foods

Look at the salt or sodium labels on food

Do not add salt at the table

Buy low salt alternatives

Buy low sodium alternatives

Do not add salt when cooking

Use spices other than salt when cooking

Avoid eating out

Others

1. B9. If you have selected "Others" in B8, please provide some examples.
2. B10. What do you think about general salt consumption in the Malaysian population? *

*Mark only one oval.*

Low

Medium

High

Don’t know

1. B11. In your understanding, what are the major food sources that are high in salt? (You * can answer more than one)

*Check all that apply.*

Processed foods

Junk foods

Fast foods

Others

1. B12. If you have selected "Others" in B11, please provide some examples.
2. B13. Do you know other alternatives or ways to increase the flavour of food without * adding more salt and other flavour enhancers?

*Mark only one oval.*

Yes

No

Not sure

1. B14. Why do you eat out? (You can answer more than one) *

*Check all that apply.*

It is convenient

To meet and mingle with friends and family

- 1. cook at home, so I eat out occasionally to escape the kitchen

It is cost-effective/cheaper

I don’t have a choice. E.g. no cooking facility at home

1. B15. Have you attempted to reduce your salt intake in the foods that you purchased or * during eating out?

*Mark only one oval.*

Yes

No

1. B16. If yes to B15, how did you attempt to reduce your salt intake in the foods that you buy, cook, or during eating out? (You can answer more than one)

*Check all that apply.*

- 1. select dishes with low-salt version from the menu

I select food items I buy with lower salt

I request cooks to reduce the salt content in my meals

I adjust the saltiness of purchased foods once at home

I do not add or use other sauces e.g. soy sauce, tomato sauce

I do not add salt in my cooking

1. B17. When buying packaged food items, how often do you look for the nutrition * information?

*Mark only one oval.*

Never

Rarely

Sometimes

Often

Always

1. B18. Do you specifically look for the salt content in nutritional labels? *

*Mark only one oval.*

Yes

No

1. B19. Do you think it would be helpful to have information about the salt content on food * packages?

*Mark only one oval.*

Yes

No

Not sure

1. B20. Do you think the salt reduction campaigns and messages promoted by the Ministry * of Health (MOH) are efficient?

*Mark only one oval.*

Yes

No

Not sure

1. B21. What would make it difficult for you to choose less salt when eating out? *

*Check all that apply.*

Low salt version to choose from not available

Requests for reduced salt in meals are not met

No control over the amount of salt used by cooks

1. B22. How do you think reducing the salt content in food and beverages will affect you? * (You can answer more than one)

*Check all that apply.*

It will reduce the taste and appeal

It will reduce the creaminess and mouthfeel

- 1. will eat less

I will not buy the low content versions

1. B23. What do you think about the availability of low-salt food products in the market? *

*Mark only one oval.*

Common

Rare/not common

I am not aware that there are low-salt food products

1. B24. Do you agree that low-salt food products in the market are more expensive? *

*Mark only one oval.*

Yes

No

Don’t know

1. B25. Which do you think is/are important factor(s) when promoting salt reduction in the * foods that you purchased or during eating out? (You can answer more than one)

*Check all that apply.*

Promoting health as the ultimate goal

Dissemination of knowledge on the methods of salt reduction

Having effective communication channels, e.g. social media, talks for community residences,

TV and radio advertisements, food festivals that feature reduced-salt food options

Targeting younger consumers

Make available more low salt foods to buy

Tell us how to cook low salt food

Make low salt food cheaper

Make compulsory for food sellers reduce the salt in the food they serve

Make compulsory for food manufacturers to reduce the salt content in the foods

48. B26. How do you think the government should be involved in promoting salt reduction in * the foods that you purchase or during eating out? (You can answer more than one)

*Check all that apply.*

Promotion and recognition of premises selling healthier foods e.g. low salt foods

Control over regular salt sales and distribution

Enforcement of mandatory lower salt content in manufactured food

Enforce mandatory lower salt served by restaurants and food vendors

Provide subsidies or cheaper salt substitutes for manufactured food products

Have salt content displayed on food labels

Increase affordability of low-salt food products

Have salt tax

Regulation of street food vendors by monitoring and enforcement of salt content

Only allow low salt foods to be sold in school canteen

Only allow low salt foods to be sold in all government premises

# Part C: Questionnaire on KAP towards sugar-sweetened beverages (SSB) intake and sugar tax

The questions in this section aim to determine knowledge, attitudes and practices towards SSBs.

There are no right or wrong answers, and we value your answers with credibility and transparency.

49. Please answer yes or no/agree or disagree to the following statements. *

| **No.** | **Items** | **Yes** | **No** |
| --- | --- | --- | --- |
| 1. | WHO recommends reducing the intake of simple sugar to less than 10% of total energy intake per day. |  |  |
| 2. | Intake of SSB is considered an appropriate strategy for increasing caloric intake in individuals with inadequate energy intake. |  |  |
| 3. | Dextrose is the scientific name of simple sugar. |  |  |
| 4. | 15 g (1 tbsp) of sugar is equivalent to 15 g of carbohydrate in our diet. |  |  |
| 5. | Calories provided by 1 tbsp of condensed milk is equivalent to half tbsp of sugar. |  |  |
| 6. | A 250ml can of Energy Drink (Red Bull) is equivalent to 6.5 teaspoons of sugar. |  |  |
| 7. | Beverages with corn syrup stated in the list of ingredients classified as SSB. |  |  |
| 8. | SSB includes beverages with added honey. |  |  |
| 9. | Fresh fruit juices are classified as SSB. |  |  |
| 10 | Plain chocolate drinks are classified as SSB. |  |  |
| 11. | Frequent consumption of SSBs increases the risk of obesity. |  |  |
| 12. | Frequent consumption of SSBs increases the risk of developing diabetes. |  |  |
| 13. | Frequent consumption of SSBs increases the risk of tooth decay. |  |  |
| 14. | Severe obese increases the risk of developing diabetes. |  |  |
| 15. | Severe obese increases the risk of heart disease. |  |  |
| 16. | Severe obese increases the risk of developing asthma. |  |  |
| 17. | Severe obese increases the risk of developing high blood pressure. |  |  |
| 18. | Severe obese increases the risk of developing cancer. |  |  |
| **No.** | **Items** | **Agree** | **Disagree** |
| 1. | It is important to read the list of the ingredients before choosing packed beverages in the market. |  |  |
| 2. | Consumers must know on how to read the label of foods or beverages especially with added sugar. |  |  |
| 3. | Choosing beverages without added sugar is much healthier compared to SSB. |  |  |
| 4. | Consumer should aware of other names of sugar that often added in food. |  |  |
| 5. | I need to have a good knowledge on reading the nutrition facts before choosing or buying foods or beverages in the market. |  |  |
| 6. | Consumers should know the health consequences of excessive consumption of SSBs such as obesity, diabetes, high blood pressure, heart disease and cancer. |  |  |
| **No.** | **Items** | **Yes** | **No** |
| 1. | I often identify the amount of sugar added in my drinks before consuming. |  |  |
| 2. | I often select flavoured milk compared to fresh milk. |  |  |
| 3. | I often compare the calorie contributed by the sugar added for each beverage before buying the item |  |  |
| 4. | I usually consumed SSBs only for breakfast. |  |  |
| 5. | I usually consume SSBs with fast food. |  |  |
| 6. | I usually consume 3 In 1 beverage as it is easy to prepare and convenient for my daily schedule. |  |  |
| 7. | I usually drink SSBs while (watching TV, cinema, playing electronic games or studying) |  |  |

In 2019, an added tax of 40 sen per litre for SSBs containing 5 grams of sugar per 100 ml and fruit juices with 12 grams of sugar per 100 ml. The tax is further increased to 50 sen per litre recently in 2024.

1. C32. What did you do when the SSBs tax was introduced? *

*Mark only one oval.*

Replace it with non-taxable drinks (such as fresh fruit juices, water, or unflavored milk)

Reduce consumption of SSBs (such as soft drinks and energy drinks).

There is no change at all.

1. C33. Indicate your agreement that you are in favour of the Malaysian SSBs tax. *

*Mark only one oval.*

Strongly disagree

Disagree

Neutral

Agree

Strongly agree

1. C34. From the options provided below, what would you most prefer the money * generated from the sugar tax be used for?

*Mark only one oval.*

Fundamental obesity interventions

Revenue for the government

Subsidizing healthy foods

Researching cures for diseases exacerbated by excess sugar consumption

# PART D: Questionnaire on KAP towards Diabetes Mellitus

Please select the most appropriate option.

1. D1. Do you usually do at least 30 min of daily physical activity at work and/or during * leisure time?

*Mark only one oval.*

Yes

No

1. D2. How often do you eat vegetables, fruit or berries? *

*Mark only one oval.*

Everyday

Not everyday

1. D3. **(For females only)** Have you ever been found to have gestational diabetes (Diabetes in pregnancy)?

*Mark only one oval.*

Yes

No

1. D4. **(For females only)** If Yes to D3, at which age?
2. D5. Have you been on any medication for diabetes, e.g., metformin or insulin? *

*Mark only one oval.*

Yes

No

1. D6. Have you or any other members of your immediate family or other relatives been * diagnosed with diabetes?

*Mark only one oval.*

No

Yes: grandparent, aunt, uncle or rst cousin

Yes: parent, brother, sister or own child

Don’t know

1. D7. Diabetes mellitus is a condition of … *

*Mark only one oval.*

High blood pressure

High blood sugar

High cholesterol

All of the above

1. D8. Type 2 Diabetes… *

*Mark only one oval.*

Is contagious

All patients can be cured

Is a progressive disease that can lead to a lot of complications

Is a short term disease

1. D9. The risk factors of Type 2 Diabetes are as follows: *

*Mark only one oval.*

Family history of diabetes

Eating too much sugar and other sweet foods

Overweight

All of the above

1. D10. “Hypoglycemia” means… *

*Mark only one oval.*

High blood pressure

Low blood pressure

High blood sugar

Low blood sugar

1. D11. Which of the following is/are (a) symptom(s) of low blood sugar? *

*Mark only one oval.*

Giddiness

Sweating

Shakiness

All of the above

1. D12. Which of the following is a symptom of high blood sugar (hyperglycemia)? *

*Mark only one oval.*

Always thirsty

Frequent urination

Sudden loss of weight

All of the above

1. D13. In long term, uncontrolled diabetes can lead to *

*Mark only one oval.*

Kidney failure

Leg amputation

Blindness

Heart attack

Stroke

All of the above

1. D14.The insulin that has been used should be stored in… *

*Mark only one oval.*

The refrigerator

The freezer

The car

A cool, dry place

1. D15.The best site for an insulin injection is… *

*Mark only one oval.*

The abdomen

The thighs

The buttocks

All of the above

1. D16. Insulin should always be injected in the same spot… *

*Mark only one oval.*

True

False

1. D17. When you travel, your medications and supplies should be… *

*Mark only one oval.*

Checked in your luggage

Carried with you

Left at home

1. D18. When you become ill and unable to eat, you should do the following EXCEPT *

*Mark only one oval.*

Immediately stop taking your medications

Drink more fluids

Monitor blood sugar frequently

Continue to take your medications

1. D19. Traditional medicines and herbal products are able to cure diabetes completely *

*Mark only one oval.*

True

False

1. D20. If you experience unwanted side effects from your medications, you should… *

*Mark only one oval.*

Tell your pharmacists or doctors about the side effects

Throw away the medications

Continue taking the medications

Give the medications to your friend that use the same medications

1. D21. The following food is converted to sugar in the body EXCEPT *

*Mark only one oval.*

Bread

Biscuits

Rice

Green leafy vegetables

1. D22. Fruits can be taken as much as you like because they contain natural sugar *

*Mark only one oval.*

True

False

1. D23. Regular exercise *

*Mark only one oval.*

Improves cholesterol levels

Strengthens the heart

Lower blood sugar

Does all of the above

1. D24. A glycosylated hemoglobin (HbA1c) test measures blood sugar over the past *

*Mark only one oval.*

Hour

Day

Week

- 1. to 3 months

1. D25. Which should NOT be used to treat low blood sugar? *

*Mark only one oval.*

Half cup of orange juice

Sweets

Eat 1 tablespoon of sugar

One cup of diet soft drink

1. D26. The following are targets for good blood sugar control EXCEPT *

*Mark only one oval.*

HbA1c < 7%

Fasting blood sugar 4.4 to 7.0 mmol/L

Blood sugar immediately after meal 2.0 to 10.0 mmol/L

- 1. hours after meal blood sugar 4.4 to 8.5 mmol/L
