# Supplementary material for: Reduction of Salt and Sugar Contents in Canteen Foods and Intakes By Students and Staff at a Malaysian Higher Education Institution: Protocol for a Mixed Methods Study
Source: JMIR Res Protoc. 2025 Jul 21;14:e69610. doi: 10.2196/69610 (PMC12322606; doi:10.2196/69610)
Supplement: Multimedia Appendix 6 [file resprot_v14i1e69610_app6.docx]

**PATIENT INFORMATION SHEET**

**Study Title: Knowledge, Attitude, Practice (KAP), Perceptions, Barriers and Enablers (PBE) of Salt, Oil and Sugar (SOS) Usage and Intake Among Sunway Campus Canteen Staff**

**Ethics Board Approval Number:** **SUREC 2024/040**

**We would like to invite you to take part in a research study. Before you decide whether to participate, you need to understand why the research is being done and what it would involve. Please take time to read the following information carefully; talk to others about the study if you wish.**

**Feel free to ask any member of the study team if there is anything that is not clear or if you would like more information. Take time to decide whether or not you wish to take part.**

**1. What is the purpose of this study?**

This study aims to assess to evaluate the Knowledge, Attitude, Practice (KAP) and Perceptions, Barriers, and Enablers (PBE) of Sunway University and Sunway College canteen staff about salt, oil, and sugar, in conjunction with the ongoing "Less Salt Less Oil Less Sugar" campaign by the Sunway Campus Facilities Services/Campus with a Conscience.

**2. Why is this study important?**

College canteens are places for centralized dining. One crucial stage in developing healthy personal habits is during higher education, where the diets of college students who have left their family environment also undergo changes that can impact their health in the short and long term. However, the intake of oil, salt, and sugar is related to the individual’s eating behavior and the canteen staff’s health literacy and cooking behavior, which can supply a healthy eating environment. Individual behavior change is more likely to be facilitated and sustained if the environment within which choices are made supports healthy food options. As part of the nutrition and healthy canteen evaluation system, this questionnaire will help assess the KAP and PBE of canteen staff and guide future intervention studies and nutritional and healthy canteen construction.

**3. What type of study is this?**

It is a cross-sectional study on the KAP and PBE towards salt, oil, and sugar usage and intake.

**4. What is the procedure that is being tested? (If applicable)**

This study does not involve the testing of any procedures/ products.

**5. Does the investigatory product contain culturally sensitive ingredients e.g.: bovine or porcine? (if applicable)**

This study does not involve the testing of any procedures/ products.

**6. Why have I been invited to participate in this study?**

We invite all individuals 18 years and above working as canteen staff at Sunway University or Sunway College to participate.

**7. Who should not participate in the study?**

Individuals below 18 years old and who are unable to give informed consent should not participate in this study.

**8. Can I refuse to take part in the study?**

Yes, you are allowed to do so as this study is entirely voluntary.

**9. What will happen to me if I take part?**

The study is a cross-sectional study where you will be required to answer a series of questionnaires on KAP and PBE towards salt, oil, and sugar usage and intake.

**10. How long will I be involved in this study?**

The study requires approximately 20 minutes.

**11. What are the possible disadvantages and risks?**

Participants will not face any major disadvantages or risks in this study as the study only involves the answering of the questionnaire.

**12. What are the possible benefits to me?**

The findings of the study could be used as ways to increase awareness of reducing salt, oil, and sugar usage and intake at a workplace/studyplace.

**13. Who will have access to my medical records and research data?**

The researchers directly involved in this study will have access to the data collected in this study. Nevertheless, all measurements will be anonymized, and it will not be possible to link any of the data back to specific individuals.

**14. Will my records/data be kept confidential?**

All data collection will be anonymized, coded, and stored electronically in Sunway University in a password-protected file. The hard copy of the data will be kept in a locked cabinet in the Department of Medical Sciences.

**15. What will happen if I don’t want to carry on with the study?**

If you decide not to participate in the study, you may drop out voluntarily.

**16. What happens when the research study stops?**

The study team will analyze the data of the research study. No further interventions or contact will be made with the study participants.

**17. What will happen to the results of the research study?**

Data from the study will be analyzed, and the results of the study may be published in an academic journal and/or distributed through other media channels.

**18. Will I receive compensation for participating in this study?**

You will be compensated with RM 10 per person for the successful completion of the questionnaire.

**19. Who should I contact if I have additional questions/problems during the course of the study?**

Should you have any concerns about this study, you may contact Sunway University Research Ethics Committee at: researchethics@sunway.edu.my.

You may also contact the Principal Investigator of the study, Prof Dr Chia Yook Chin, Head of Department of Medical Sciences, School of Medical & Life Sciences, Sunway University at ycchia@sunway.edu.my

**Study Title:** **Knowledge, Attitude, Practice (KAP), Perceptions, Barriers and Enablers (PBE) of Salt, Oil and Sugar (SOS) Usage and Intake Among Sunway Campus Canteen Staff**

| Date of survey | |  |
| --- | --- | --- |
| **Part A: Participants Details** | | |
| 1. Age | | (years) |
| 2. Gender | |  Male  Female |
| 3. Nationality | | - Malaysian - Non-Malaysian. Please state: __________ |
| 4. Ethnicity | |  Malay   Chinese   Indian  Others. Please state: ________________ |
| 5. | Please select your **highest** educational level (do NOT consider the education level you are currently undertaking). | - No formal education - Primary - Secondary - Tertiary - Pre-university/foundation/diploma - Undergraduate - Postgraduate |
| 6. | Working experience | - 1 year - 1 - 3 years - 3 – 10 years - ≥10 years |
| **Part B: Questionnaire on KAP towards salt and sugar usage and intake** | | |
|  | Have you ever heard of the Vendor SOS "Less Salt Less Oil Less Sugar" campaign? | - Yes - No |
|  | How much salt we should eat per day that is recommended by the World Health Organisation (WHO)? | - ≤ 5 g/day - ≤ 6 g/day - ≤ 9 g/day - ≤ 12 g/day - I don’t know |
|  | What are the harmful effects of excessive salt intake on human health? (You can answer more than one) | - Can lead to high blood pressure. - High salt intake is a risk factor for stroke - High salt intake is a risk factor for heart failure - Increases the risk of gastritis and stomach cancer. - Increases the risk of kidney diseases. - Increases the risk of osteoporosis. |
|  | Where do you get the information about the relationship between salt and blood pressure? | - From pamphlets or publicity column - Broadcast, TV - Newspapers, magazines - Meetings or symposium - Doctors - Nurses - Dieticians - Pharmacists - Friends - Family - Others:_________ - I don’t know |
|  | High salt intake may have a great effect on blood pressure. It is actually the sodium in salt that plays the key role. True or false? | - True - False - I don’t know. |
|  | Which of the following foods are high in salt? (You can answer more than one) | - Street foods, e.g. fish balls, nuggets - Fermented dishes, e.g. budu, cincalok, tempoyak - Staple Malaysian dishes, e.g. asam pedas, fried noodles, noodle soup, etc. - Traditional food, e.g. belacan (shrimp paste), ikan bilis (dried anchovies), dried shrimp, salted fish - Prawn crackers/fish crackers - Canned foods (e.g. canned soups, canned sardines, canned tuna) - Poppadum - Bread - Others: ___________________ |
|  | How much fat and oil intake is recommended to adults in the Malaysian Dietary Guidelines? | - 10-20% of total calories/day - 20-30% of total calories/day - 30-40% of total calories/day - I don’t know. |
|  | Which of the following statements is true? (You can answer more than one) | - High intake of animal source fats such as lard and butter is not good for health. - Compared with animal source fats, all of the plant source oils are good for health. - Tropical vegetable oils such as coconut oil and palm oil are rich in saturated fatty acid and are good for health over a long period of time. - Cod liver oil is good for cardiovascular health. |
|  | Which of the following foods are rich in fat/oil? | - Fried food - Sandwich biscuit, shortbread, cookies - Cream cake - Chocolate - Instant noodles - Mayonnaise |
|  | What are the harmful effects of high fat/oil intake on human health? (You can answer more than one) | - Can lead to obesity - Can lead to fatty liver disease - Can lead to high cholesterol in blood - Can increase risk of heart attacks - May have some effects on gastrointestinal function - Long-term high fat/oil intake can lead/contribute to brittle bones (osteoporosis) - Long-term high fat/oil intake may affect endocrine and reproductive functions. |
|  | What kind of sugar should be reduced? | - Fructose - The sugar in sweet foods - The intake of carbohydrates such as bread, rice, potatoes as these foods can converted to sugar in our body - Added sugar |
|  | How much sugar is recommended for adults in the Malaysian Dietary Guidelines? | - Less than 25 g/day - Less than 50 g/day - Less than 75 g/day - I don’t know. |
|  | Which of the following are actually added sugar? (You can answer more than one) | - Sucrose - Fructose - Glucose - Table sugar - Xylitol - Cyclamate, Aspartame |
|  | Do you think which of the following foods usually contain added sugars? (You can answer more than one) | - Cakes and biscuits - Cola, Sprite, and other carbonated drinks - Fruit juices - Yogurt drinks - Packet and bottle drinks such as soybean milk, honey grapefruit tea, etc. - Some dishes such as sweet and sour fish, sweet soup, etc. - Red bean paste, mung bean paste, and other fillings. |
|  | What are the harmful effects of high sugar intake on human health? | - Can lead to overweight and obesity - Can lead to dental caries - Can lead to or worsen diabetes - Affects calcium absorption - Can lead to heart attack - Makes gout worse - Increases the risk of short-sightedness/myopia if excessive intake of sugar for a long time. |
|  | What is the fasting blood sugar to diagnose diabetes? | - ≥ 5.0 mmol/L - ≥ 7.0 mmol/L - ≥ 9.0 mmol/L - I don’t know |
|  | Which of the following drinks are suitable for drinking in daily life? | - Plain water or tea - Sugary coffee - Fruit and vegetable juice - Milk tea - Functional beverages like probiotic drinks, dietary fibre drinks, etc. - Carbonated drinks |
|  | What are the measures to reduce sugar intake in daily life? (You can answer more than one) | - Replace sugar with syrup - Replace sugar with honey - Use low-sugar recipes - Replace sugar with sweeteners that meet appropriate safety standards - Reduce the intake of tomato sauce, barbecue sauce, dried fruit and plum |
|  | What's the low-sugar limit? | - ≤2.5g/100g (or 100ml) - ≤5g/100g (or100ml) - I don’t know |
|  | What is the sugar-free standard? | - ≤0.5g/100g (or 100ml) - Sugar-free refers to foods without added sugar - I don’t know |
|  | Do you think the dishes sold in this canteen are salty? | - Yes - Just right - No - Don’t know; I don’t consume foods sold on campus |
|  | Do you think the dishes you sell in this canteen is salty? | - Yes - Just right - No |
|  | Do you agree to reduce the amount of salt in the food you sell in the canteen? | - Yes, I agree - No, I do not agree - No, as my food is not salty - Not sure |
|  | Do you plan to reduce the amount of salt you use after you know the bad effects of eating too much salt? | - Yes - No - I am not sure |
|  | What do you think are the main difficulties you have in trying to reduce the amount of salt in the food you sell in the canteen? (You can answer more than one) | - Salt reduction in dishes will affect the taste. - Salt reduction is not necessary. - Buyers will not accept/find my food to be tasty with reduced salt - I will lose sales if I reduce salt in my foods as it will be less tasty - Because of lack of appropriate measuring tools, it is difficult to control the amount of salt used. - I do not know how to make my food tasty without using enough salt - Other ingredients in place of salt that can make food tasty is not readily available/on sale - Other replacement/substitutes for salt is expensive - A variety of salt seasonings was used during cooking, there is no standard process. - Others: ________________________ |
|  | What factors do you think affect the promotion of salt reduction measures in canteens? (You can answer more than one) | - Lack of information/knowledge on how to reduce salt without affecting the taste or sale of foods - No direction/guide on how much salt we need to reduce - Lack of funds to support it. - It will not be popular with teachers and students - Lack of support from management department and government policy - Lack of help from the professionals - Others: ___________________________ |
|  | Do you think the dishes in this canteen is greasy? | - Yes - Just right - No - Don’t know; I don’t consume foods sold on campus |
|  | Do you think it is necessary to reduce the use of cooking oil/fat in this canteen? | - Very necessary - Necessary - Not necessary - Not sure |
|  | If conditions permit, are you willing to use a spoon for a fixed amount of oil when cooking? | - Yes - No - Not sure |
|  | Do you agree to reduce the amount of cooking oil/fat used in the cooking process? | - Very agree - Agree - Not agree - Not sure |
|  | What do you think are the main difficulties affecting the implementation of oil reduction measures? | - Lack of information/knowledge on how to reduce oil without affecting the taste or sale of foods - No direction/guide on how much oil we need to reduce - Lack of funds to support it. - It will be not popular with teachers and students - Lack of support from management department and government policy - Lack of help from the professionals - Others: _________________________ |
|  | What do you think about the amount of sugar that is eaten in daily life by Malaysians? | - Very high - A little high - Just right - Malaysians should eat less or none at all - Others: _____________________________ |
|  | What do you think of the sweetness of the sugary food in this canteen? | - Very sweet - Just right - Not really sweet - Don’t know; I don’t consume foods sold on campus |
|  | Are you in favor of a low-sugar diet? | - Very agree - Agree - Not agree - Not sure |
|  | Do you agree that we should drink more boiled water instead of sugary drinks? | - Very agree - Agree - Not agree - Not sure |
|  | From your point of view, what do you think is the reason for the reluctance to reduce sugar? (You can answer more than one) | - Personal eating preferences - Eating sweets can help reduce stress. - Eating sweets does not have a major impact on one's health - Eating sweets and drinking beverages is a fashionable lifestyle - Others: _____________________________ |
|  | What do you think are the main difficulties affecting the implementation of sugar reduction measures? (You can answer more than one) | - Sugar reduction during cooking affects taste. - Sugar reduction is not necessary. - Because of lack of appropriate measuring tools, it is difficult to control the amount of sugar used. - No standard process, the use of sugar is determined by the chef. - There is no difficulty |
|  | Do you think it is necessary to reduce the use of oil, salt, and sugar in this canteen？ | - Very necessary - Necessary - Not necessary - Not sure |
|  | Do you think it is necessary to implement “low SOS” dishes? | - Very necessary - Necessary - Not necessary - Not sure |
|  | What difficulties do you think in setting up a “low SOS” in this canteen? | - I will lose sales - Lack of funds to support it - It is not popular with teachers and students - Lack of support from management department and government policy - Lack of the help from the professionals - Others: ­­­­­­­_____________ |
|  | Do you think reducing salt, sugar, and oil is equally important for health? | - Yes, all of them are equally important. - Salt reduction is more important. - Sugar reduction is more important. - Oil reduction is more important. - None of them is important. |
|  | When you cook, how do you control the amount of salt? | - Pour out directly from the bottle. - Use a quantitative salt spoon. - Use a tablespoon (The exact amount of salt in a spoon is not sure.) - Others： __________________ |
|  | In the last two months, how often have you used a rationed salt spoon? | - Always - Often - Sometimes - Occasionally - Never |
|  | When do you usually put salt in your cooking? | - At the beginning - In the middle - At the end of cooking - Not fixed, any time |
|  | How often do you eat salty foods with high salt content, such as picked vegetables and salted duck eggs？ | - Always - Often - Sometimes - Occasionally - Never |
|  | What methods have you used to reduce the amount of salt? | - Use quantitative salt spoon - Use low-sodium salt. - Use vinegar, lemon juice, sugar, to flavor the dish instead of salt. - Use pepper, garlic, hot pepper to flavor the dish. - Use salt-free mixed spices, such as thirteen spices, etc. to flavor - Use soy sauce, soybean paste, etc. to replace salt. - No method has not been used |
|  | When you buy packaged food, do you pay attention to the salt/sodium content in the food? | - Always - Often - Sometimes - Occasionally - Never |
|  | When you cook, how do you control the amount of oil? | - By experience - Using a tablespoon - Use a graduated oil jug - Others: _______________ |
|  | How often do you cook with animal source fat? | - Always - Often - Sometimes - Occasionally - Never |
|  | When shopping for packaged foods, how often do you read the Nutrition Facts Labels to choose low-in-fat or free of trans fatty acids foods? | - Always - Often - Sometimes - Occasionally - Never |
|  | How often do you eat fatty foods? Such as fried food, cookies or biscuits, cream cake, chocolate, instant noodles, mayonnaise and so on. | - Eat everyday - Often - Sometimes - Occasionally - Never |
|  | What methods have you taken to reduce the amount of oil used in cooking? (You can answer more than one) | - Cooking by steaming, boiling, braising and cold dressing instead of frying. - Deliberately reduce the use of oil when cooking. - Use a measuring device such as an oil pot to control the amount of oil used - Any measure has not been taken to reduce oil |
|  | How often do you drink leftover dishes and soup when you eat at home? | - Eat everyday - Often - Sometimes - Occasionally - Never |
|  | How often do you eat processed foods with added sugar, such as cake, cookies, ice cream and preserved fruit? | - Eat everyday - Often - Sometimes - Occasionally - Never |
|  | Which of the following drinks would you like to drink when you are thirsty? | - Water or tea - Carbonated drinks - Fruit and vegetable juice - Milk tea - Functional beverages - Sweetened coffee |
|  | What kind of food do you add sugar to? (You can answer more than one) (You can answer more than one) | - Soy Milk - Coffee - Porridge - Tea - Tofufah - I don’t use sugar |
|  | How often do you measure added sugars with a scale or other measuring instruments when making desserts? | - Always - Often - Sometimes - Occasionally - Never - I have never made desserts |
|  | Do you consciously reduce the intake of high-sugar foods in your daily diet? | - Yes, I do pay more attention to it. - Yes, I pay attention to it. - Neutral - No, I don’t pay much attention to it. - No, I never pay attention to it. |
|  | What is your staple food? | - Rice or flour - Rice or flour mainly, and often eat whole grain - Rice or flour mainly, occasionally whole grain - Whole grain flour mainly, occasionally rice or flour - Always whole grains, sometimes rice or flour - Others:____________ |
|  | When buying packaged foods, how often do you choose low-sugar or sugar-free foods by reading the nutrition facts list? | - Always - Often - Sometimes - Occasionally - Never |
| The following questions are some of the possible **barriers** faced by you when reducing salt and sugar during food preparation, Please choose how much you disagree or agree to the following statements. | | |
|  | There is lack of standard guidelines on how much to reduce salt or sugar. | - Strongly disagree - Disagree - Neutral - Agree - Strongly agree |
|  | There is lack of awareness from the consumers on the importance of salt and sugar reduction. | - Strongly disagree - Disagree - Neutral - Agree - Strongly agree |
|  | There is concern that the customers will not accept or will complain if the foods are not salty or sweet enough. | - Strongly disagree - Disagree - Neutral - Agree - Strongly agree |
|  | There is lack of consumer requests for reduced salt and sugar. | - Strongly disagree - Disagree - Neutral - Agree - Strongly agree |
|  | Salt/sugar reduction is not possible as that will negatively affect the taste, quality and creaminess of the foods and beverages. | - Strongly disagree - Disagree - Neutral - Agree - Strongly agree |
|  | I do not wish to reduce salt/sugar in traditional food or heritage recipes as it might alter the taste and compromise such food’s customary and authentic flavours. | - Strongly disagree - Disagree - Neutral - Agree - Strongly agree |
|  | Regular salt is much cheaper than salt substitutes such as potassium chloride and natural flavour enhancers such as dry and fresh herbs and spices. | - Strongly disagree - Disagree - Neutral - Agree - Strongly agree |
|  | There is lack of rules and regulations to guide the preparation of foods with reduced salt and sugar. | - Strongly disagree - Disagree - Neutral - Agree - Strongly agree |
| The following questions are some of the possible **enablers** faced by you when reducing salt and sugar during food preparation. Please choose how much you disagree or agree to the following statements. | | |
|  | It is important to have a standard guideline for salt and sugar reduction in food. | - Strongly disagree - Disagree - Neutral - Agree - Strongly agree |
|  | Salt and sugar reduction courses could be integrated into the existing mandatory Food Handling Course. | - Strongly disagree - Disagree - Neutral - Agree - Strongly agree |
|  | There should be comprehensive knowledge  and guidance from government research agencies such as MARDI on the preparation of foods with reduced salt and sugar without compromising the taste and sensory appeal. | - Strongly disagree - Disagree - Neutral - Agree - Strongly agree |
|  | Maintaining good health and reducing medical  expenses are strong incentives for active participation in the salt and sugar reduction policy. | - Strongly disagree - Disagree - Neutral - Agree - Strongly agree |
|  | It is important to educate consumers on the importance and methods of salt and sugar reduction in foods. | - Strongly disagree - Disagree - Neutral - Agree - Strongly agree |
|  | Awareness of salt and sugar intake and their presence in foods must commence at the school level. | - Strongly disagree - Disagree - Neutral - Agree - Strongly agree |
|  | For the customer to accept salt and sugar reduction, there must be gradual reduction in salt content without their knowledge.  In addition, they recommended presenting salt  separately on tables | - Strongly disagree - Disagree - Neutral - Agree - Strongly agree |
|  | For the customer to accept salt and sugar reduction, salt and sugar should be presented separately on tables for them to add themselves. | - Strongly disagree - Disagree - Neutral - Agree - Strongly agree |
|  | I would introduce a reduced salt or reduced sugar menu at my stall. | - Strongly disagree - Disagree - Neutral - Agree - Strongly agree |
|  | There should be promotion and recognition of health-conscious food premises by the government, e.g. Healthy Choice Stall/  Caterer,’ and star-rating system. | - Strongly disagree - Disagree - Neutral - Agree - Strongly agree |
|  | Food packaging labels should be regulated to indicate high-salt food products. | - Strongly disagree - Disagree - Neutral - Agree - Strongly agree |
|  | Distribution of regular salt and high-salt products should be controlled. | - Strongly disagree - Disagree - Neutral - Agree - Strongly agree |
|  | Price of regular salt should be regulated and taxed to discourage excessive salt usage. | - Strongly disagree - Disagree - Neutral - Agree - Strongly agree |
|  | The price of natural flavour enhancers and salt substitutes should be decreased to encourage more their more extensive usage and reduce reliance on conventional salt. | - Strongly disagree - Disagree - Neutral - Agree - Strongly agree |
|  | The Ministry of Health and local authorities should collaborate in monitoring and regulating the salt and sugar contents in foods and their usage when preparing foods. | - Strongly disagree - Disagree - Neutral - Agree - Strongly agree |
