# Supplementary material for: Reduction of Salt and Sugar Contents in Canteen Foods and Intakes By Students and Staff at a Malaysian Higher Education Institution: Protocol for a Mixed Methods Study
Source: JMIR Res Protoc. 2025 Jul 21;14:e69610. doi: 10.2196/69610 (PMC12322606; doi:10.2196/69610)
Supplement: Multimedia Appendix 7 [file resprot_v14i1e69610_app7.docx]

| **ONLY SELECTED PARTICIPANTS ARE INVITED TO PARTICIPATE IN THIS PART** |
| --- |

**Study Part 3: 6-month interventional study - exposure to foods sold on campus that have reduced salt and sugar**

**BASELINE (START OF STUDY)**

| **Participant ID:** |  |
| --- | --- |
| **Email address:** |  |
| **Date:** |  |

| **Part A. Blood pressures, anthropometric and body composition measurements**  *These measurements will be taken for you.* |
| --- |

| **Measurement** | **1^st^ Reading** | **2^nd^ Reading** |
| --- | --- | --- |
| Systolic Blood Pressure, SBP (mmHg) |  |  |
| Diastolic Blood Pressure, DBP (mmHg) |  |  |
| Pulse rate, PR (bpm) |  |  |
| Waist circumference (cm) |  |  |
| Hip circumference (cm) |  |  |
| Height (cm) |  |  |
| Weight (kg) |  |  |
| Total Body Fat, TBF (%) |  |  |
| Visceral Fat Level, VFL (%) |  |  |
| Resting Metabolism, RM (kcal) |  |  |
| Body Mass Index, BMI (kg/m^2^) |  |  |
| Body age |  |  |
| ***Area check*** | **Subcutaneous Fat, SF (%)** | **Skeletal Muscle, SM (%)** |
| Whole body |  |  |
| Trunk |  |  |
| Arms |  |  |
| Legs |  |  |

| **Cardiovascular Health** | | | |
| --- | --- | --- | --- |
| **Classification** | **SBP (mmHg)** | **DBP (mmHg)** | **PR (a.k.a. Heart Rate)** |
| Normal | <120 | and < 80 | 60 – 100 bpm |
| Prehypertension | 120-139 | or 80-89 |  |
| Stage 1 hypertension | 140-159 | or 90-99 |  |
| Stage 2 hypertension | ≥160 | or ≥100 |  |

| **Obesity Indicators** | | | |
| --- | --- | --- | --- |
| **Classification** | **BMI (kg/m^2^)** | **Waist Circumference (cm)** | |
|  |  | **Men** | **Women** |
| Underweight | < 18.5 |  |  |
| Normal | 18.5-22.9 | <90 | <80 |
| Overweight | 23.0 – 27.5 |  |  |
| Obese | ≥ 27.5 | ≥90 | ≥80 |
| **Classification** | **VFL (%)** | **TBF (%)** | |
|  |  | **Men** | **Women** |
| - (Low) | - | 5.0-9.9 | 5.0-19.9 |
| 0 (Normal) | 0.5-9.5 | 10.0-19.9 | 20.0-29.9 |
| + (High) | 10.0-14.5 | 20.0-24.9 | 30.0-34.9 |
| ++ (Very high) | 15.0-30.0 | 25.0-50.0 | 35.0-50.0 |
| **Classification** |  | **SM (%)** | |
|  |  | **Men** | **Women** |
| - (Low) |  | 5.0-32.8 | 5.0-25.8 |
| 0 (Normal) |  | 32.9-35.7 | 25.9-27.9 |
| + (High) |  | 35.8-37.3 | 28.0-29.0 |
| ++ (Very high) |  | 37.4-60.0 | 29.1-60.0 |

| **Part B: Two Weekday One Weekend 24-hour diet record**   - Please write down all foods and beverages consumed for three 24-hour time periods. Each day starting at 12:00 am and ending at 11:59 pm. Choose three consecutive days, including **two weekdays** and **one weekend**. - List the approximate **Time** the meal was consumed, **Place** where it was consumed (home, campus, name of restaurant, *pasar malam*, etc.), and the type of eating occasion or **Meal** (breakfast, lunch, dinner, snack, or other). - List each **Food/Beverage Item** you consumed, including foods eaten between meals and all drinks, even if it is a non-caloric item like water, coffee, tea, or sugar free gum. - Specify **Details/Ingredients/Preparation/Brand Name** of each food or beverage consumed. **Pay attention also to the sauce or gravy in the food.** - Record the **Amount** of each food or beverage consumed. Portion sizes can be recorded in a variety of ways, please use the method that works best for you. You can use the appended “**Food Portion Size**” to help you document portion sizes. Portion sizes can be recorded using the following standard measurements: - Solid foods – use volume in cups, tablespoons or teaspoons - Weight in grams - Liquids – use volume in ml - Fraction of the whole (e.g. 1/8 of 9" pie) |
| --- |

**Food Portion Size**

******

***Example***

| ***Time*** | ***Place*** | ***Meal*** | ***Food/beverages*** | ***Details/Ingredients/Method of Preparation -(baked, fried, boiled, canned etc.)/Brand Name*** | ***Amount/Serving Size*** |
| --- | --- | --- | --- | --- | --- |
| *6.30 am* | *Home cooked* | *Breakfast* | - *Fried mee* | - *Mee* - *Chicken* - *Tomato sauce* - *Chilli sauce* - *Oyster sauce* - *Salt* - *Carrot* | - *1 cup* - *1 ½ cup* - *1 tbspn* - *1 tbspn* - *½ tspn* - *1 tsp (4 serving)* - *1 tbspn* |
| *10.00 am* | *Office – self brought* | *Snack* | - *Milo* - *Biscuit cream* - *crackers* | - *Milo 3 in 1* - *Biscuit Hup Seng* | - *1 packet* - *3 pieces* |
| *1.00 pm* | *Fresco Sunway University* | *Lunch* | - *Rice* - *Fried Fish* - *Vegetables soup* | - *White rice* - *Mackerel fish* - *Carrot cabbage* | - *1 cup* - *1 piece* - *1 small bowl* |
| *6.30 pm* | *Rock Café Sunway City* | *Dinner* | - *Rice* - *Fried chicken* - *Sawi* - *Curry gravy only* | - *White rice* - *Drumstick* - *Stir-fried with sambal belacan* - *Curry gravy cooked with chicken and potato* | - *1 cup* - *1 medium piece* - *½ cup* - *1 ladle* |
| *11.30 pm* | *Home cooked* | *Supper* | - *Maggi mee soup* | - *2-minute Maggi instant mee curry flavor* - *Chicken Egg* | - *1 packet* - *1 large (Grade A)* |

**Weekday 1 (Please mention the specific day: ___________)**

| **Time** | **Place** | **Meal** | **Food/beverages** | **Details/Ingredients/Method of Preparation - baked, fried, boiled, canned etc./Brand Name** | **Amount/Serving Size** |
| --- | --- | --- | --- | --- | --- |
|  |  |  |  |  |  |
|  |  |  |  |  |  |
|  |  |  |  |  |  |
|  |  |  |  |  |  |
|  |  |  |  |  |  |
|  |  |  |  |  |  |
|  |  |  |  |  |  |
|  |  |  |  |  |  |
|  |  |  |  |  |  |
|  |  |  |  |  |  |
|  |  |  |  |  |  |
|  |  |  |  |  |  |
|  |  |  |  |  |  |
|  |  |  |  |  |  |
|  |  |  |  |  |  |
|  |  |  |  |  |  |

**Weekday 2 (Please mention the specific day: ___________)**

| **Time** | **Place** | **Meal** | **Food/beverages** | **Details/Ingredients/Method of Preparation - baked, fried, boiled, canned etc./Brand Name** | **Amount/Serving Size** |
| --- | --- | --- | --- | --- | --- |
|  |  |  |  |  |  |
|  |  |  |  |  |  |
|  |  |  |  |  |  |
|  |  |  |  |  |  |
|  |  |  |  |  |  |
|  |  |  |  |  |  |
|  |  |  |  |  |  |
|  |  |  |  |  |  |
|  |  |  |  |  |  |
|  |  |  |  |  |  |
|  |  |  |  |  |  |
|  |  |  |  |  |  |
|  |  |  |  |  |  |
|  |  |  |  |  |  |
|  |  |  |  |  |  |

**Weekend (Please mention the specific day: ____________)**

| **Time** | **Place** | **Meal** | **Food/beverages** | **Details/Ingredients/Method of Preparation - baked, fried, boiled, canned etc./Brand Name** | **Amount/Serving Size** |
| --- | --- | --- | --- | --- | --- |
|  |  |  |  |  |  |
|  |  |  |  |  |  |
|  |  |  |  |  |  |
|  |  |  |  |  |  |
|  |  |  |  |  |  |
|  |  |  |  |  |  |
|  |  |  |  |  |  |
|  |  |  |  |  |  |
|  |  |  |  |  |  |
|  |  |  |  |  |  |
|  |  |  |  |  |  |
|  |  |  |  |  |  |
|  |  |  |  |  |  |
|  |  |  |  |  |  |

| **3. Collection of urine samples**  *Instruction*: **TWO** urine collection containers with your participant ID will be given to you on the day this form is given out as shown below:  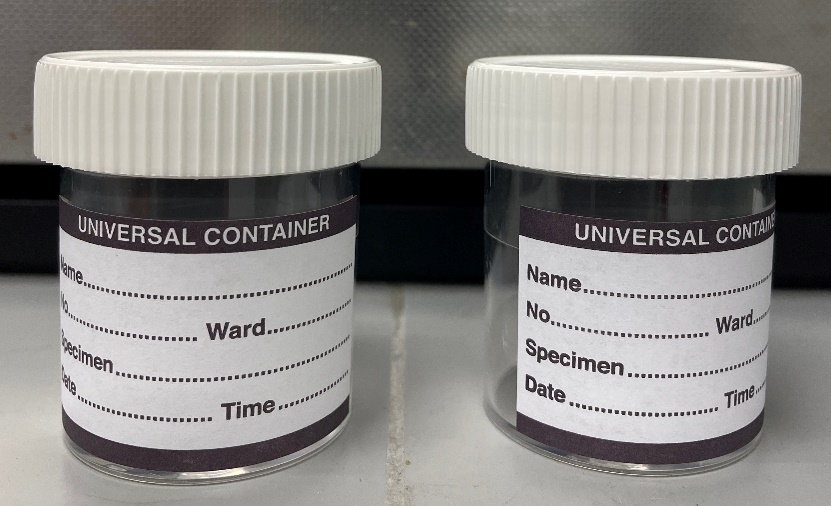  Please collect your urine at the following times:   1. **First passage of urine upon waking up in the morning.** 2. **Before you leave the campus for home at late afternoon or early evening.**   **Tips on collecting urine:**   - 1. Cleanse the urinary opening. Please wipe or rinse your external genitals.   2. Begin to urinate into the toilet.   3. Pass the collection container into your urine stream.   4. Fill up at least half of the collection container.   5. Finish urinating into the toilet. |
| --- |

| **ONLY PARTICIPANTS WITH ID “XYZ” NEED TO FILL THIS PART** |
| --- |

| **4. Saltiness Intensity Perception and Pleasantness Rating of Selected Dish on Campus**  *Instruction*: Please mark a horizontal line on the following scale on how you perceive the **intensity of saltiness** and how much do you **like or dislike** any **ONE** selected dish that you have tasted on campus. There is no correct or wrong answer for this. |
| --- |

| Stall name: |  | |
| --- | --- | --- |
| Dish name: |  | |
| Description of dish: |  | |
| **Saltiness Intensity Perception** | | **Pleasantness Rating** |
| 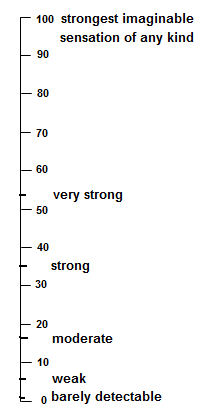 | | 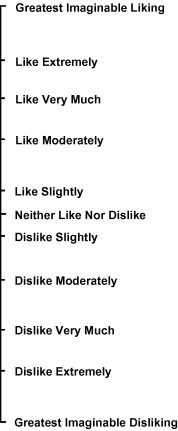 |
| Photograph of dish: |  | |
| Salt content of dish: (to be filled up by researcher) |  | |

| **END OF STUDY. YOU WILL BE GIVEN A RM 10 VOUCHER UPON COMPLETION OF ALL PARTS IN THIS FORM. WE WILL CONTACT YOU FOR A FOLLOW-UP 3 MONTHS FROM THE DATE OF THIS VISIT. THANK YOU.** |
| --- |
