# Supplementary material for: Reduction of Salt and Sugar Contents in Canteen Foods and Intakes By Students and Staff at a Malaysian Higher Education Institution: Protocol for a Mixed Methods Study
Source: JMIR Res Protoc. 2025 Jul 21;14:e69610. doi: 10.2196/69610 (PMC12322606; doi:10.2196/69610)
Supplement: Multimedia Appendix 2 [file resprot_v14i1e69610_app2.pdf]

4 April 2024

Ref No: SUREC 2024/029

Prof. Datin Dr Chia Yook Chin
Department of Medical Sciences
School of Medical and Life Sciences (SMLS)
Sunway University

Dear Prof. Datin Dr Chia Yook Chin,

Research Ethics Approval of "An Interventional Study of Salt and Sugar Reduction in Foods Sold On Campus at Sunway University and Sunway College"

Sunway University Research Ethics Committee has reviewed the documents you have submitted for your project and has given **approval** for your proposed study.

The final list of documents reviewed and approved by the Committee is as per below:

1. Ethics Application Form
2. Participation Information Sheet
3. Consent Form
4. Questionnaires
5. Citi Report

Please inform the Committee of any changes in your research scope and/or timeline.

The Committee wishes you success in your research project.

Yours sincerely,

Prof. Mahendhiran Sanggaran Nair
Chair,
Sunway University Research Ethics Committee

Sunway University
Research Ethics Committee
Research Ethics Approval Application

Title of project: An Interventional Study of Salt and Sugar Reduction in Foods Sold On Campus at Sunway University and Sunway College

Principal Investigator: Prof. Datin Dr. Chia Yook Chin (Department of Medical Sciences, School of Medical & Life Sciences, Sunway University)

School: School of Medical and Life Sciences

Co-Investigators:
1. Dr. Say Yee How (Department of Biological Sciences, School of Medical and Life Sciences, Sunway University)
2. Dr Cheng Maong Hui (Department of Medical Sciences, School of Medical and Life Sciences, Sunway University)
3. Assoc. Prof. Dr Felicia Chung (Department of Medical Sciences, School of Medical and Life Sciences, Sunway University)
4. Dr Lau Tze Pheng (Department of Biological Sciences, School of Medical and Life Sciences, Sunway University)
5. Assoc. Prof. Dr Ooi Pei Boon (Department of Medical Sciences, School of Medical and Life Sciences, Sunway University)
6. Dr. Nurul Husna Shafie (Department of Nutrition, Faculty of Medicine and Health Sciences, Universiti Putra Malaysia)

Duration of project (including planned end date): 2 years (1 April 2024 – 30 March 2026)

Research category:

~~Arts & Applied arts~~	Clinical & Health sciences	~~Information & Communication Technology~~	~~Natural & Cultural Heritage~~	~~Pure & Applied Science~~	~~Social Sciences~~	~~Technology & Engineering~~

Materials included application		Please tick
1	CITI certificate	✓
2	Participant Information Sheet	✓
3	Consent form	✓
4	Questionnaires	✓
5	Interview outlines	N/A
6	Biosafety approval	N/A
7	Animal Welfare Proforma	N/A
8	Other materials (specify):	N/A

Brief outline of the project.	University students and staff members on campus, especially those who frequently eat out and spend considerable time on campus, form a crucial demographic facing challenges related to high salt and sugar intake in out-of-home food. Recognizing the prevalence of this lifestyle among these populations, it becomes imperative to understand the perceptions and experiences of both students and campus communities regarding salt and sugar consumption. Furthermore, serving lowered salt foods at workplaces have been shown to be able to reduce salt intake by at least 0.5 g/day.

	In this study, we aim to shed light on the dynamics of the university campus food environment, where students and campus communities heavily depend on vendors for their meals. The insights gained will not only benefit the individuals who regularly eat out but will also contribute to creating a healthier food landscape on university campuses, impacting the overall well-being of the individuals. This study holds significance in addressing the shared responsibility between individuals who use out-of-home food and vendors in promoting a healthier lifestyle within the university community.<br><br>Thus, this study aims to assess the effects of salt and sugar reduction in foods sold on campus at Sunway University and Sunway College. For the first part of the study, the knowledge, attitude, and practice (KAP) of dietary salt and sugar intake among Sunway University and Sunway College students and staff will be assessed. For the second part of the study, selected participants will be invited to participate in an interventional study for 6 months, where they will be exposed to foods sold on campus that have reduced salt and sugar.
Please describe the research methodology explaining items such as,<br>1. **detailing the procedures for recruiting participants**,<br>2. sources of data &/or research samples,<br>3. methods of data collection & archiving,<br>4. storage and disposal of any waste,<br>5. compensation to participants, etc.	**1. Procedures/Sources of data and/or research samples**<br>*Study Part 1*: An online validated questionnaire measuring the knowledge, awareness, and practices (KAP) of dietary salt and sugar intake. It will be open to the entire Sunway community, and the community has the right not to do the survey or withdraw at any point.<br>The questionnaire will assess :<br>1. Socio-demographic and lifestyle factors – questions such as age, gender, ethnicity, medical history, smoking and drinking practices<br>2. KAP towards dietary salt and sugar intakes, and diabetes<br>3. Barriers and enablers for salt reduction<br><br>**Inclusion and exclusion criteria**<br>*Inclusion criteria*<br>1. Students and staff of Sunway University and Sunway College aged 18 years and above<br>2. Able to provide informed consent (verbal/written) to participate<br>3. Understands English<br><br>*Exclusion criteria*<br>1. Individuals with acute illness, psychological or psychiatric conditions<br><br>*Study Part 2*: An interventional study for 6 months will expose them to foods sold on campus that have reduced salt and sugar. A total of 150 participants who eat often foods sold on campus will be selected from Study Part 1 in a randomized manner and they form the intervention group who will be eating the foods sold on campus that have reduced salt and sugar. Another 150 participants who do not eat often the food sold on campus will be the control group.

	Participants will need to provide urine samples for the measurement of urinary sodium, potassium, chloride, and creatinine, have body composition and anthropometric measurements taken, perform 24-hour dietary records, perform saltiness intensity perception and pleasantness ratings of the foods on campus, at three timepoints - at the start of the study, after 3 months and after 6 months.<br><br>**Inclusion and exclusion criteria**<br>To meet the 0.5 g difference in sodium (salt), the sample size required will be 150 per arm.<br><br>*Inclusion criteria for intervention group*<br>1. Individuals who eat on campus food for at least 3 meals per week<br>2. Consent to participate<br><br>*Inclusion criteria for control Group*<br>1. Individuals who do not eat on campus food or for <3 meals per week<br>2. Consent to participate<br><br>*Exclusion criteria*<br>Individuals with acute illness, psychological or psychiatric conditions<br><br>Participants would need to provide an identifiable name, contact number, and email address in the informed consent form, in order for them to be contactable for follow-up in Part 2 of the study. All participants will still be assigned with a participant number.<br><br>Part 2 will only be conducted once part 1 has recruited a comfortable amount of participants, e.g. 500 people. This is to enable selection based on inclusion and exclusion criteria as stated in the application form. Those who fulfill these criteria will be assigned into intervention and control groups. Selection will be done randomly based on the participant number through statistical software or online random number generator.<br><br>**2. Data storage and archiving**<br>Questionnaire data will be stored and archived in a cloud storage Google Drive and external hard disk as a backup, for at least 5 years before they are deleted.<br><br>**3. Compensation to participants**<br>1. For KAP participants: No compensation but they are entitled to enter a lucky draw to win three lucky draw food hampers.<br>2. For intervention study participants: They will be given RM 10 for each successful completion of questionnaire, dietary records, anthropometric and body composition measurements, and providing urine samples at three

	timepoints - at the start of the study, after 3 months and after 6 months (maximum RM 30 per participant).

Questions		Y/N	Details
1	Does the research involve human subjects (including answering questionnaires)?	Y	Human subjects will be recruited from Sunway University and Sunway College.
2	Does the research involve children or other vulnerable groups?	N	N/A
3	Have you obtained other materials such as archives or other research data?	N	N/A
4	Do you know of any ethics or legal issues involving your research partner, external funder or collaborator?<br>If yes, please explain.	N	N/A
If biomedical research, please answer below			
1	Does the research involve non-human animal subjects?	N	N/A
2	Does the research involve the use of human or animal materials?	Y	Intervention and control participants will provide two spot urine samples - first morning void and last void in the evening before leaving campus, at three timepoints - at the start of the study, after 3 months and after 6 months. The participants will be given urine containers for them to collect the urine samples themselves, to be placed in a ziplock bag provided to them too. These samples are to be placed in a basket placed outside the Student Project Lab at Level 4. These samples will be collected by a personnel from the Laboratory Department of Sunway Medical Centre for further processing.
3	Does the research involve the use of any bio-hazardous materials, such as infectious agents?	N	N/A
4	Do you have biosafety approval? Please provide approval letter in application.	N	N/A
5	How are you disposing of your materials?	Y	Urine samples will be processed and any remaining urine waste will be discarded away by Sunway Medical Centre
