# Supplementary material for: Reduction of Salt and Sugar Contents in Canteen Foods and Intakes By Students and Staff at a Malaysian Higher Education Institution: Protocol for a Mixed Methods Study
Source: JMIR Res Protoc. 2025 Jul 21;14:e69610. doi: 10.2196/69610 (PMC12322606; doi:10.2196/69610)
Supplement: Multimedia Appendix 3 [file resprot_v14i1e69610_app3.pdf]

SUNWAY UNIVERSITY

8 May 2024

Ref No: SUREC 2024/040

Prof. Datin Dr Chia Yook Chin
Department of Medical Sciences
School of Medical and Life Sciences (SMLS)
Sunway University

Dear Prof. Datin Dr Chia Yook Chin,

Research Ethics Approval of "Knowledge, Attitude, Practice (KAP), Perceptions, Barriers and Enablers (PBE) of Salt, Oil and Sugar (SOS) Usage and Intake Among Sunway Campus Canteen Staff"

Sunway University Research Ethics Committee has reviewed the documents you have submitted for your project and has given **approval** for your proposed study.

The final list of documents reviewed and approved by the Committee is as per below:

1. Ethics Application Form
2. Participation Information Sheet
3. Consent Form
4. Questionnaires
5. Citi Report

Please inform the Committee of any changes in your research scope and/or timeline.

The Committee wishes you success in your research project.

Yours sincerely,

Prof. Mahendhiran Sanggaran Nair
Chair,
Sunway University Research Ethics Committee

Sunway University
Research Ethics Committee
Research Ethics Approval Application

Title of project: Knowledge, Attitude, Practice (KAP), Perceptions, Barriers and Enablers (PBE) of Salt, Oil and Sugar (SOS) Usage and Intake Among Sunway Campus Canteen Staff

Principal Investigator: Prof. Datin Dr. Chia Yook Chin (Department of Medical Sciences, School of Medical & Life Sciences, Sunway University)

School: School of Medical and Life Sciences

Co-Investigators:
1. Dr. Say Yee How (Department of Biological Sciences, School of Medical and Life Sciences, Sunway University)
2. Dr Cheng Maong Hui (Department of Medical Sciences, School of Medical and Life Sciences, Sunway University)
3. Assoc. Prof. Dr Felicia Chung (Department of Medical Sciences, School of Medical and Life Sciences, Sunway University)
4. Dr Lau Tze Pheng (Department of Biological Sciences, School of Medical and Life Sciences, Sunway University)
5. Assoc. Prof. Dr Ooi Pei Boon (Department of Medical Sciences, School of Medical and Life Sciences, Sunway University)

Duration of project (including planned end date): 2 years (1 April 2024 – 30 March 2026)

Research category:

~~Arts & Applied arts~~	Clinical & Health sciences	~~Information & Communication Technology~~	~~Natural & Cultural Heritage~~	~~Pure & Applied Science~~	~~Social Sciences~~	~~Technology & Engineering~~

Materials included application		Please tick
1	CITI certificate	✓
2	Participant Information Sheet	✓
3	Consent form	✓
4	Questionnaires	✓
5	Interview outlines	N/A
6	Biosafety approval	N/A
7	Animal Welfare Proforma	N/A
8	Other materials (specify):	N/A

Brief outline of the project.	Excess cooking oil and salt use in catering services contributes to obesity and cardiovascular diseases. College canteens are places for centralized dining. One crucial stage in developing healthy personal habits is during higher education, where the diets of college students who have left their family environment also undergo changes that can impact their health in the short and long term. However, the intake of oil, salt and sugar is related to the individual's eating behavior and the canteen staff's health literacy and cooking behavior, which can supply a healthy eating environment. Individual behavior change is more likely to be facilitated and sustained if the environment within which choices are made supports healthy food options.

	Therefore, evaluating the knowledge, attitude, practice (KAP), perceptions, barriers and enablers (PBE) of canteen staff about oil, salt and sugar usage and intake is very important.<br><br>Thus, the primary aim of this study is to evaluate the KAP and PBE of Sunway University and Sunway College (collectively known as Sunway Campus) canteen staff about oil, salt, and sugar, in conjunction with the ongoing "Less Salt Less Oil Less Sugar" campaign by the Sunway Campus Facilities Services/Campus with a Conscience. As part of the nutrition and healthy canteen evaluation system, this questionnaire will help assess the KAP and PBE of canteen staff and guide future intervention studies and nutritional and healthy canteen construction.
Please describe the research methodology explaining items such as,<br>1. **detailing the procedures for recruiting participants**,<br>2. sources of data &/or research samples,<br>3. methods of data collection & archiving,<br>4. storage and disposal of any waste,<br>5. compensation to participants, etc.	**<u>1. Procedures/Sources of data and/or research samples</u>**<br>A pen-and-paper validated questionnaire measuring the KAP and PBE of salt, oil and sugar usage and intake. It will be open to the entire Sunway Campus canteen staff, and the staff has the right not to do the survey or withdraw at any point.<br>The questionnaire will assess :<br>1. Socio-demographics– questions such as age, gender, ethnicity<br>2. KAP towards salt, oil and sugar usage and intake<br>3. PBE towards salt, oil and sugar usage and intake<br><br>**Inclusion and exclusion criteria**<br>*Inclusion criteria*<br>1. Sunway Campus canteen staff aged 18 years and above<br>2. Able to provide informed consent (verbal/written) to participate<br>3. Understands English<br><br>*Exclusion criteria*<br>1. Individuals with acute illness, psychological or psychiatric conditions<br><br>**<u>2. Data storage and archiving</u>**<br>Questionnaire data will be stored and archived in a cloud storage Google Drive and external hard disk as a backup, for at least 5 years before they are deleted.<br><br>**<u>3. Compensation to participants</u>**<br>They will be given RM 10 for each successful completion of questionnaire. It will be given on the spot to the participants who have completed the pen-and-paper questionnaire.

Questions		Y/N	Details
1	Does the research involve human subjects (including answering questionnaires)?	Y	Human subjects will be recruited from Sunway University and Sunway College.
2	Does the research involve children or other vulnerable groups?	N	N/A

3	Have you obtained other materials such as archives or other research data?	N	N/A
4	Do you know of any ethics or legal issues involving your research partner, external funder or collaborator?<br>If yes, please explain.	N	N/A
If biomedical research, please answer below			
1	Does the research involve non-human animal subjects?	N	N/A
2	Does the research involve the use of human or animal materials?	N	N/A
3	Does the research involve the use of any bio-hazardous materials, such as infectious agents?	N	N/A
4	Do you have biosafety approval? Please provide approval letter in application.	N	N/A
5	How are you disposing of your materials?	N	N/A
