# Supplementary material for: Reduction of Salt and Sugar Contents in Canteen Foods and Intakes By Students and Staff at a Malaysian Higher Education Institution: Protocol for a Mixed Methods Study
Source: JMIR Res Protoc. 2025 Jul 21;14:e69610. doi: 10.2196/69610 (PMC12322606; doi:10.2196/69610)
Supplement: Multimedia Appendix 4 [file resprot_v14i1e69610_app4.pdf]

MALAYSIAN SOCIETY OF HYPERTENSION
(Persatuan Hipertensi Malaysia)
The Secretariat
A Menarini Spore Pte Ltd
B-18-2, Level 18, The Ascent Paradigm,
No.1, Jalan SS7/26A, Kelana Jaya,
47301 Petaling Jaya, Selangor Darul Ehsan, Malaysia
Tel: 03 – 79857000 | msh.secretariat.m@gmail.com
www.msh.my

2023 / 2025 Council Members

President
Dato' Sri Dr. Azhari Rosman

Vice-President
Prof. Dr. Nik Sherina Hanafi

Hon General Secretary
Dato' Wira Dr. Chandran

Hon Treasurer
Assoc. Prof. Dr. Ching Siew Mooi

Council Members
Prof. Datin Dr. Chia Yook Chin Dr. Chong Kuck Meng
Prof. Dr. Khoo Ee Ming
Dr. Navin Kumar A/L Devaraj
Dr. Beh Hooi Chin
Dr. Iskandar Mirza

Dr Daniel Cheng Maong Hui
Department of Medical Sciences,
School of Medical & Life Sciences,
Sunway University

28th May 2024

Dear Dr Daniel Cheng Maong Hui

RE: MSH Grant Review Committee Response on Proposal Application Titled "An Interventional Study of Salt and Sugar Reduction in Foods Sold On a University/College Campus in Malaysia"

The MSH Grant review committee has reviewed your proposal. There are few comments as attached in Appendix 1 that they would like to have responses to before a final decision is made. Please respond by 4 June so that the committee can review again and present this in the coming council meeting on 10 June.

Thank you.

Yours Sincerely,

Honorary Professor Dr Ee Ming Khoo
MBBS (London) MD (Mal) MRCGP(UK) FAMM FAFP
Department of Primary Care Medicine
Faculty of Medicine
University of Malaya
50603 Kuala Lumpur
MALAYSIA.
Tel: +603 79492306
Fax: +603 79577941
khooem@um.edu.my

MALAYSIAN SOCIETY OF HYPERTENSION
(Persatuan Hipertensi Malaysia)
The Secretariat
A Menarini Spore Pte Ltd
B-18-2, Level 18, The Ascent Paradigm,
No.1, Jalan SS7/26A, Kelana Jaya,
47301 Petaling Jaya, Selangor Darul Ehsan, Malaysia
Tel: 03 – 79857000 | msh.secretariat.m@gmail.com
www.msh.my

2023 / 2025 Council Members

President
Dato' Sri Dr. Azhari Rosman

Vice-President
Prof. Dr. Nik Sherina Hanafi

Hon General Secretary
Dato' Wira Dr. Chandran

Hon Treasurer
Assoc. Prof. Dr. Ching Siew Mooi

Council Members
Prof. Datin Dr. Chia Yook Chin
Dr. Chong Kuck Meng
Prof. Dr. Khoo Ee Ming
Dr. Navin Kumar A/L Devaraj
Dr. Beh Hooi Chin
Dr. Iskandar Mirza

Appendix 1 – Feedback from reviewers

Please add study duration: start date/month and end date/month

Part 3 study B. Do all the food vendors include vendors who sell beverages? How about vendors who might not have control over their food ie pre-cooked meals

There is a possibility that food with reduced salt and sugar might not appeal to potential customers, thus the food vendors face a risk of a drop in sales. What are the investigators' plans to ensure that all the food vendors comply with the reduction strategies?

Please describe selection and recruitment methods of the participants.

The definition of intervention vs control group in terms of 3 meal/week. Is this based on evidence or not? Any references? This opens to variability on face value.

Are participants supposed to eat the 'intervention food' throughout the six months period? How would investigators account for holiday periods when participants might not be on campus thus dietary intake might be compromised.

Is there a specific time that the urine samples need to be submitted in relation to the timing of eating?

Budget: Maximum budget allowed is RM20,000. Please revise. Please take note that token for participants is limited up to RM5 per participant for quantitative study. There is different information given in the budget on food sample analysis. In the budget, 6 food samples x 20 vendors was written but in Part 3, Study A, 5 samples for 17 vendors. Please amend which is the correct version

Dr. Daniel Cheng Maong Hui
Department of Medical Sciences,
School of Medical & Life Sciences,
Sunway University,
5, Jalan Universiti,
47500 Bandar Sunway,
Selangor.

2nd June 2024

Honorary Professor Dr Ee Ming Khoo
MBBS (London) MD (Mal) MRCGP(UK) FAMM FAFP
Department of Primary Care Medicine
Faculty of Medicine
University of Malaya
50603 Kuala Lumpur.

Re: Reviewers Feedback and Response

We thank the reviewers for their valuable responses. We have addressed them here and we have attached the amended proposal herewith.

Please add study duration: start date/month and end date/month
Study duration added: 1 June 2024 – 31 May 2025

Part 3 study B. Do all the food vendors include vendors who sell beverages? How about vendors who might not have control over their food ie pre-cooked meals
Proposal has been amended to salt reduction only in Part 3 of study. This will exclude beverages. We are aware that some vendors will not have control over reducing salt contents, i.e. pre-cooked/supplied meals. This information will be included in the first objective of Part 3 Study A.

There is a possibility that food with reduced salt and sugar might not appeal to potential customers, thus the food vendors face a risk of a drop in sales. What are the investigators' plans to ensure that all the food vendors comply with the reduction strategies?
An executive order by the top management (Facilities & Services) to lower salt will be implemented. Vendors will be met and briefed on this order and the potential benefits of it (like gaining new health-conscious customers). However, participation is voluntary and no penalty will be implemented.

Please describe selection and recruitment methods of the participants.
Selection of participants for Part 1 will be via convenience sampling. Recruitment method will be via email blast to students and staff, and promotional banners and distribution of leaflets around campus. Selection of participants for Part 3 will be via random sampling. They will be invited via email to participate in this part of the study.

The definition of intervention vs control group in terms of 3 meal/week. Is this based on evidence or not? Any references? This opens to variability on face value.
The number of meals/week as stated in the proposal, which has been amended to 5, is just an estimation. An average number of meals/week will be calculated and will be used as a cutoff point to determine intervention vs control groups.

Are participants supposed to eat the 'intervention food' throughout the six months period? How would investigators account for holiday periods when participants might not be on campus thus dietary intake might be compromised.
Yes, participants in the intervention group will eat the 'intervention food' with reduced salt throughout the six months period. Participants will consist of staff and students of Sunway University and Sunway College. Both institutions have different semester systems, with non-overlapping mid-semester and between-semester breaks. Students might be away during the semester breaks, but not simultaneously for both institutions. Staff will remain on campus unless they go for a long holiday break. Therefore, intervention participants will have a good mixture of those who will eat consistently on campus.

Is there a specific time that the urine samples need to be submitted in relation to the timing of eating?
We are unable to dictate the exact times the participants should eat and collect urine. Therefore, urine collection is directed as follows: first morning void and last void in the late afternoon or early evening, prior to the evening meal.

Budget: Maximum budget allowed is RM20,000. Please revise. Please take note that token for participants is limited up to RM5 per participant for quantitative study. There is different information given in the budget on food sample analysis. In the budget, 6 food samples x 20 vendors was written but in Part 3, Study A, 5 samples for 17 vendors. Please amend which is the correct version
Budget revised to maximum RM 20,000. Token for participants for Part 3 (funded by MSH) has been amended to RM 5. Food sample analysis budget has been amended accordingly. A targeted 10 food samples per vendor for 17 vendors – taken at baseline and 3 months after invention will yield a total number of 340 samples. RM 30 per sample x 340 = RM 10,200.

With these feedbacks and amendments, it is our great hope that our proposal will be accepted.

Thank you.

Yours sincerely,

Dr. Daniel Cheng Maong Hui
Senior Lecturer
Department of Medical Sciences
School of Medical and Life Sciences
Sunway University
Email: danielcmh@sunway.edu.my
Tel: +603-74918622

Title:
An Interventional Study of Salt and Sugar Reduction in Foods Sold On a University/College Campus in Malaysia

Investigators:

No	Name	Affiliation
1.	Dr. Cheng Maong Hui (Principal Investigator)	Department of Medical Sciences, School of Medical & Sciences, Sunway
2.	Professor Datin Dr Chia Yook Chin (Co-Principal Investigator)	Department of Medical Sciences, School of Medical & Sciences, Sunway
3.	Dr. Say Yee How (Co-Investigator)	Department of Medical Sciences, School of Medical and Life Sciences, Sunway University
4.	Assoc. Prof. Dr Felicia Chung Fei Lei (Co-Investigator)	Department of Biological Sciences, School of Medical & Sciences, Sunway
5.	Dr Lau Tze Pheng (Co- Investigator)	Department of Biological Sciences, School of Medical & Sciences, Sunway
6.	Assoc. Prof. Dr Ooi Pei Boon (Co-Investigator)	Department of Medical Sciences, School of Medical & Sciences, Sunway

Study Duration: 1 June 2024 – 31 May 2025

Research Background:
Cardiovascular disease stands as a significant global health challenge, ranking among the primary causes of premature death and disability, especially in developing countries. The burden of cardiovascular disease persists as the primary cause of death in Malaysia.

In Malaysia, high blood pressure (BP) has consistently emerged as a leading contributor to premature mortality. The 2019 National Health and Morbidity Survey (NHMS) revealed a 30% prevalence of hypertension among Malaysian adults aged 18 and above. Alarmingly, despite the prevalence increasing with age, nearly two-thirds of hypertensive individuals remain undiagnosed (1).

Research indicates that a mere 2mmHg rise in systolic blood pressure (BP) significantly elevates mortality rates from stroke and ischemic heart disease in adults. Research has also shown the association of salt/sodium intake to BP and that the reduction in salt/sodium intake is also associated with lower BPs and low CV deaths.

The mean intake of salt in Malaysia is 7.9gm/day much higher than that recommended by WHO of <5 gm /day (2). In fact, the WHO has set the reduction of population salt intake by 30% by 2025.

According to the Malaysian Adult Nutrition Survey (MANS 2014), 70% of Malaysians regularly dine out, especially in urban areas and Peninsular Malaysia (3). In 2019, household spending on out-of-home foods increased to 11.2% from 8.7% in 2004/2005 (4). The trend of consuming meals away from home, particularly during breakfast, has gained popularity in countries like Uganda (5) and Malaysia.

From a study conducted among Malaysians by Arifen and colleagues (6), consumers and food operators in Malaysia perceive high salt intake in the out-of-home food sector. Food operators stress the need for a comprehensive salt reduction policy involving all stakeholders. Consumers face limited awareness and knowledge, counterproductive practices among food operators, and challenges in accessing affordable low-sodium products. Food operators encounter a lack of standardized guidelines, effective enforcement, and uncooperative consumer practices. The interviews among food vendors, caterers and consumers identify barriers such as food quality and salt prices, advocating for awareness campaigns, stricter regulations for manufactured foods, and enhanced enforcement.

University students and staff members on campus, especially those who frequently eat out and spend considerable time on campus, form a crucial demographic facing challenges related to high salt and sugar intake in out-of-home food. Recognizing the prevalence of this lifestyle among these population, it becomes imperative to understand the perceptions and experiences of both students and campus communities regarding salt and sugar consumption. Furthermore, serving lowered salt foods at workplaces have been shown to be able to reduce salt intake by at least 0.5 g/day.

In this study, we aim to shed light on the dynamics of the university campus food environment, where students and campus communities heavily depend on vendors for their meals. The insights gained will not only benefit the individuals who regularly eat out but will also contribute to creating a healthier food landscape on university campuses, impacting the overall well-being of the individuals. This study holds significance in addressing the shared responsibility between individuals who use out-of-home food and vendors in promoting a healthier lifestyle within the university community.

Thus, this study aims to assess the effects of salt and sugar reduction in foods sold on campus at Sunway University and Sunway College. For the first part of the study, the knowledge, attitude, and practice (KAP) of dietary salt and sugar intake among Sunway University and Sunway College students and staff will be assessed. For the second part of the study, KAP,

barriers and enablers of salt and sugar reduction among campus canteen staff will be conducted. Concurrently, a third part of the study involves a 6-month interventional study on salt reduction in foods sold on campus. For this part of the study, firstly, the sodium contents of selected foods sold on campus will be assessed. Then, selected participants will be invited to participate in the interventional study where they will be exposed to foods sold on campus that have reduced salt.

The budget that we are requesting from MSH covers the third part of the study.

The project acknowledges the interconnectedness of diet and cardiovascular health, emphasizing the importance of promoting mindful food choices to mitigate the risks associated with high blood pressure. obesity and its related cardiovascular diseases.

Aims and objectives of the study
The overarching goal of this multi-faceted project is to assess the effects of the reduction of salt and sugar in foods sold on campus at Sunway University and Sunway College. The study comprises the following parts and their specific objectives.

1. **Part 1: KAP on dietary salt and sugar intake among Sunway University and Sunway College community (students and staff)**
    a. Assess participants' Knowledge, Attitude, and Practice (KAP) regarding salt and sugar consumption.
    b. Assess participants' Knowledge, Attitude, and Practice (KAP) regarding diabetes mellitus.
    c. Identify common barriers and enablers in reducing salt and sugar intake.

2. **Part 2: KAP, Perception, Barriers and Enablers of Salt and Sugar Reduction among Campus Canteen Staff**
    a. Investigate food vendors KAP about salt and sugar
    b. Explore perceptions of salt and sugar reduction in the foods they sell on campus.
    c. Identify barriers and enablers to reducing salt and sugar in the foods they sell
    d. Fears about reducing salt and sugar reduction in the foods they sell on campus.
    e. Propose strategies to address identified barriers and enhance enablers for reducing salt and sugar in the foods they sell.

3. **Part 3: Interventional study on salt reduction in foods sold on campus – budget to be covered by MSH grant**

    Study A: Sodium contents of selected foods sold on campus
    a. Survey and compile a comprehensive list of existing foods (excluding beverages) sold at Sunway University and Sunway College, including those that are cooked on spot and pre-cooked/supplied.
    b. Identify specific food items with high salt content for further targeted intervention.
    c. Analyse the salt content of the surveyed foods before and after an executive order by the top management to lower salt is implemented.
    d. Propose further recommendations for reducing salt content in foods sold on campus.

    Study B: Effects of salt reduction of selected foods sold on campus on the participants
    a. Investigate the dietary salt intake by dietary records and spot urine tests in a subset of participants after the intervention of salt reduction of selected foods sold on campus.
    b. Investigate the effect of the intervention of salt reduction of selected foods sold on campus on the participants' anthropometric measurements and body composition.

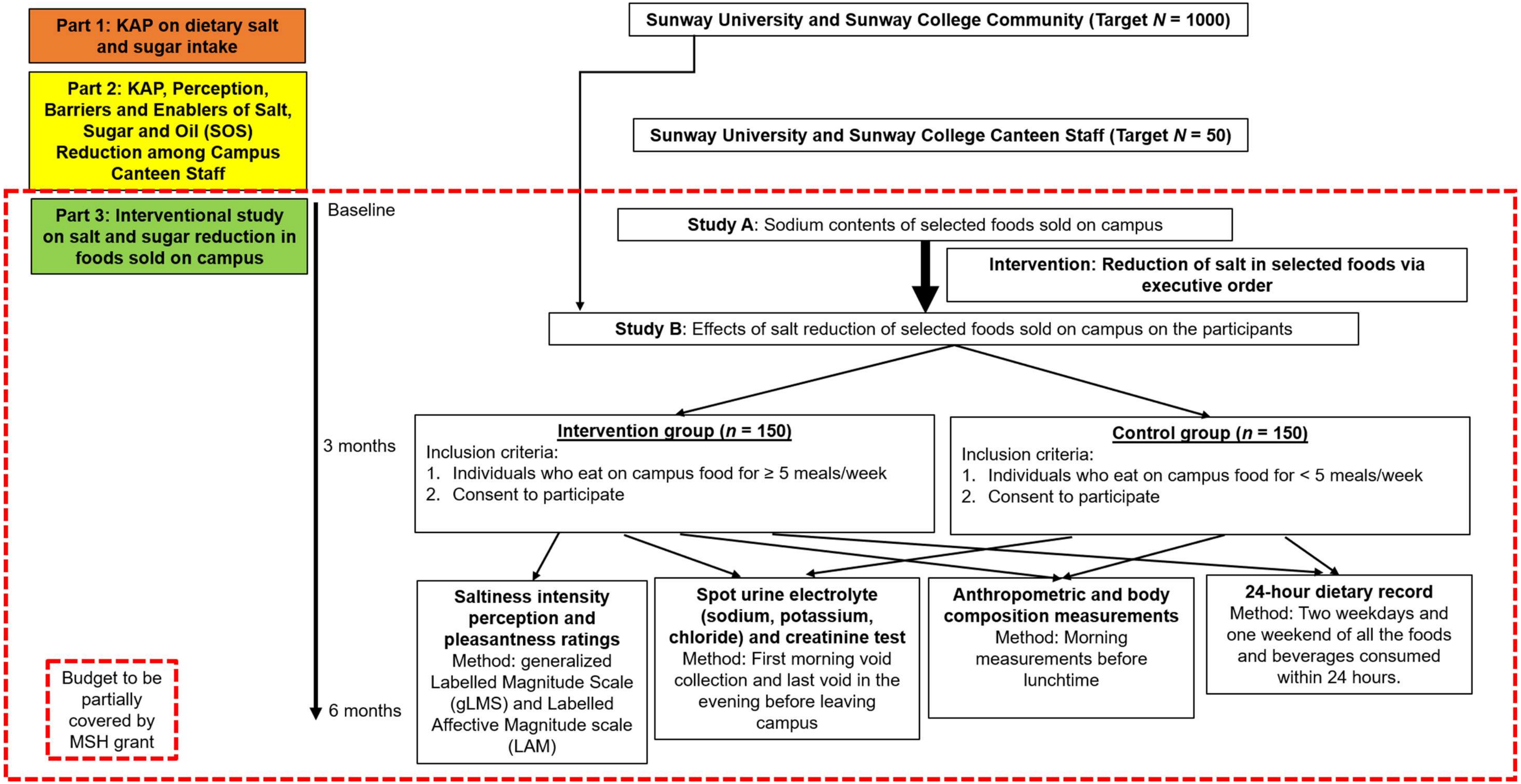

Figure 1: Diagram of the study design

Study methodology
This study will be conducted among the Sunway University and Sunway College community comprising of students and staff, at Sunway University and Sunway College, Bandar Sunway solely.

Part 1: KAP on dietary salt and sugar intake
It will be open to the entire Sunway community, and the community has the right not to do the survey or withdraw at any point. Recruitment will be by convenience sampling. Method of recruitment will be via email blast to students and staff, and promotional banners and distribution of leaflets around campus.

Sample size calculation
Using Raosoft sample size online calculator (http://www.raosoft.com/samplesize.html), a minimum sample size of 370 is needed in order to achieve a 5% margin of error, 95% confidence level, a Sunway University and Sunway College population size of around 10,000 and a 50% response distribution. Therefore, a target sample size of 1000 participants is set to take mid-study drop-outs into consideration.

Instrument
The online validated questionnaire will assess:
1. Socio-demographic and lifestyle factors – questions such as age, gender, ethnicity, medical history, smoking and alcohol-drinking practices
2. KAP towards dietary salt and sugar intakes, and diabetes
3. Barriers and enablers for salt reduction

Inclusion criteria
1. Students and staff of Sunway University and Sunway College aged 18 years and above
2. Able to provide informed consent (verbal/written) to participate
3. Understands English

Exclusion criteria
1. Individuals with acute illness, psychological or psychiatric conditions

Compensation to participants
No compensation but they are entitled to enter a lucky draw to win three lucky draw food hampers.

Part 2: KAP, Perception, Barriers and Enablers of Salt and Sugar Reduction among Campus Canteen Staff

Sample size calculation
The estimated number of campus canteen staff that are involved in food preparation is around 65 people, and given an estimate response rate of 80%, the targeted number of participants is around 50 people.

Instrument
A questionnaire on KAP, perceptions, barriers and enablers to salt and sugar reduction will be administered among the canteen staff that are involved in food preparation.

The online validated questionnaire will assess:
1. KAP towards salt and sugar usage in food preparation (including on campus) and in dietary intake
2. Perception, barriers and enablers for salt reduction during food preparation on campus

Inclusion criteria
1. Canteen staff that are involved in food preparation aged 18 years and above
2. Able to provide informed consent (verbal/written) to participate
3. Understands English

Exclusion criteria
1. Individuals with acute illness, psychological or psychiatric conditions

Compensation to participants
They will be given RM 10 for each successful completion of questionnaire.

Part 3: An interventional study for 6 months will expose them to foods sold on campus that have reduced salt.
Study A
Selected samples of food sold on campus will be analysed for their sodium contents at baseline and also 3 months after the intervention of salt reduction. Sodium level will be measured by inductively coupled plasma-optical emission spectroscopy (ICP-OES) using an outsourced commercial lab service.

A targeted 10 food samples per vendor for 17 vendors – taken at baseline and 3 months after invention will yield a total number of 340 samples.

Study B

Before the actual lowering of salt in foods sold on campus, all the food vendors will be met and briefed on this order and the potential benefits of it (like gaining new health-conscious customers). The amount of salt to be reduced for the selected foods sold and available salt alternatives will also be briefed to them. However, participation is voluntary and no penalty will be implemented.

Sample size calculation

According to WHO/PAHO Regional Expert Group for Cardiovascular Disease Prevention through Population-wide Dietary Salt Reduction Sub-group for Research and Surveillance (7), to meet the 0.5 g difference in sodium (salt), the sample size required will be 150 per arm.

Therefore, a total of 150 participants who eat often foods sold on campus will be selected from Part 1 in a randomized manner and they form the intervention group who will be eating the foods sold on campus that have reduced salt. Another 150 participants who do not eat often the food sold on campus will be the control group. They will be invited via email to participate in this part of the study. The number of meals/week as stated in the proposal is just an estimation. An average number of meals/week will be calculated and will be used as a cutoff point to determine intervention vs control groups.

Participants will need to provide urine samples for the measurement of urinary sodium, potassium, chloride, and creatinine, have body composition and anthropometric measurements taken, perform 24-hour dietary records, perform saltiness intensity perception and pleasantness ratings of the foods on campus, at three timepoints - at the start of the study, after 3 months and after 6 months.

Inclusion and exclusion criteria

Inclusion criteria for intervention group

1. Individuals who eat on campus food for at least 5 meals per week
2. Consent to participate

Inclusion criteria for control Group

1. Individuals who do not eat on campus food or for <5 meals per week
2. Consent to participate

Exclusion criteria

Individuals with acute illness, psychological or psychiatric conditions

Compensation to participants

They will be given RM 5 for each successful completion of questionnaire, dietary records, anthropometric and body composition measurements, saltiness intensity perception and pleasantness ratings, and providing urine samples at three timepoints - at the start of the study, after 3 months and after 6 months (maximum RM 15 per participant).

Instruments

Urine analysis

Intervention and control participants will provide two spot urine samples - first morning void and last void in the late afternoon or early evening, prior to the evening meal, at three timepoints - at the start of the study, after 3 months and after 6 months. This will yield a total of 300 × 2 × 3 = 1800 samples. Urine electrolytes (sodium, potassium, chloride) and creatinine will be measured using the potentiometry method (Alinity Abbott) at the laboratory department of Sunway Medical Centre, Bandar Sunway.

Assessment of saltiness intensity perception and pleasantness ratings

Participants will receive verbal and written English instructions and then completed two rating scales assessing the intensity of saltiness of the selected foods sold on campus they perceived (hereafter referred to as ‘intensity perception’), and the extent to which they liked or disliked this level of saltiness of the selected foods sold on campus (hereafter referred to as ‘pleasantness rating’). The perceived saltiness intensity will be measured by using the generalised Labelled Magnitude Scale (gLMS) (8) where the participants will be asked to make a horizontal marking on the paper versions of it. A 100 mm scale was constructed with six different descriptors which were ‘‘barely detectable’’,‘‘weak’’, ‘‘moderate’’, ‘‘strong’’, ‘‘very strong’’ and ‘‘strongest imaginable sensation of any kind’’ were spaced at 1.4 mm, 6 mm, 17 mm, 35 mm, 53 mm, and 100 mm, respectively. For pleasantness, the Labelled Affective Magnitude (LAM) scale (9) will be used. It is a 100 mm scale that is constructed with 11 different levels – ‘‘greatest imaginable disliking’’ (0 mm), ‘‘dislike extremely’’(12 mm), ‘‘dislike very much’’ (22 mm), ‘‘dislike moderately’’(34 mm), ‘‘dislike slightly’’ (45 mm), ‘‘neither like or dislike’’(50 mm), ‘‘like slightly’’ (56 mm), ‘‘like moderately’’ (68 mm), ‘‘like very much’’ (78 mm), ‘‘like extremely’’ (87 mm), ‘‘greatest imaginable liking’’ (100 mm).

Anthropometric and body composition measurements
Clinical measurements indicative of vascular health namely systolic blood pressure (SBP), diastolic blood pressure (DBP) and pulse rate will be taken using an automated blood pressure monitor (HEM-7121, Omron, Japan) after the subjects have rested for 5 min. The height, waist and hip circumferences will be measured using a measuring tape and waist-hip ratio (WHR) will be calculated by dividing waist circumference by hip circumference. A bio-impedance body composition scale (Omron HBF-375 Karada Scan) will be used to determine weight, body mass index (BMI), total body fat (TBF), visceral fat (VF), subcutaneous fat (SF), skeletal muscle percentage (SM) and resting metabolism rate (RM). The cut-off points for overweight, obesity, overall adiposity (TBF) and central adiposity (WHR) are ≥23 kg/m^2, ≥25 kg/m^2 (10), 20% (males) or 30% (females) (11) and 0.90 (males) or 0.85 (females)(12), respectively.

Data analysis
All statistical analysis will be done using the Statistical Package for Social Sciences (SPSS) version 27. The prevalence of KAP will be computed. Continuous data will be described as mean and standard deviation if the distribution is normal. However, when the data is a skewed distribution; median and interquartile range (25-75th percentiles) will be used. Categorical data will be reported as proportions (percentage). Chi-square test or Fisher exact tests will be used for the categories or dichotomous predictors. Factors associated with KAP towards intention to reduce salt and sugar intake will be determined using multiple logistic regression analysis.

The change in amount of salt and sugar reduced in food and the change in the amount of salt intake by participants will be analysed by a comparisons of means All analyses will be done with 95% confidence intervals (CI), and the level of significance will be set at p<0.05.

Ethics approval
Ethics approval will be obtained from the Ethics Committee of Sunway University.

Gantt chart
The activities of the research will be described in the Gantt chart as below.

Activities	1	2	3	4	5	6	7	8	9	10	11	12
Ethics application												
Recruitment of RA												
Development of Instrument												
Part 1 – entire Sunway University and Sunway College community												
Part 2 – campus canteen staff												
Part 3 – Study A Food analysis												
Part 3 – Study B Interventional Study												
Data analysis												
Report writing and presentation												

Budget

Items	Unit cost<br>(RM)	Total<br>(RM)
One Field Assistant allowance for 6 months, RM 10 per hour x 20 hours per week x 24 weeks	4,800.00	4,800.00
Part 3: Study A – Analysis of food samples<br>10 food samples x 17 vendors:<br>• Baseline<br>• After 3 months	 <br>30.00<br>30.00	 <br>5,100.00<br>5,100.00
Part 3: Study B<br>Token of appreciation to 300 interventional study participants<br>• Baseline<br>• After 3 months<br>• After 6 months	 <br><br><br>5.00	 <br><br><br>4,500.00
**Total**		**19,500.00**

Summary

Overall, this project aims to foster a culture of health and well-being within the Sunway University and Sunway College community while contributing to broader public health objectives and corporate social responsibility. Through collaboration between students, staff, and the institution, the project seeks to drive positive change in dietary habits, ultimately promoting a healthier lifestyle. This will help to contribute towards the WHO goals to reduce intake of salt by 30% by 2025 and towards UN's SDG goal #3 on health and well being

References

1. Institute for Public Health (IPH), National Institutes of Health. National Health and Morbidity Survey (NHMS) 2019: Vol. I: NCDs – Non-Communicable Diseases: Risk Factors and other Health Problems. Ministry of Health Malaysia; 2020.
2. Institute for Public Health (IPH), National Institutes of Health. Population-Based Salt Intake Survey To Support The National Salt Reduction Programme For Malaysia (Malaysian Community Salt Survey – MyCoSS). Ministry of Health Malaysia; 2019.
3. Institute for Public Health (IPH), National Institutes of Health. National Health and Morbidity Survey 2014: Malaysian Adult Nutrition Survey (MANS) Vol. II: Survey Findings. Ministry of Health Malaysia; 2014.
4. Department of Statistics Malaysia (DOSM). Household Expenditure Survey Report 2019 [Internet]. Department of Statistics Malaysia (DOSM); 2019. Available from: https://v1.dosm.gov.my/v1/uploads/files/1_Articles_By_Themes/Prices/HIES/HES-Report/HES_Malaysia.pdf
5. Sseguya W, Matovu N, Swann J, Draper A. Contribution of street food to dietary intake of habitual urban consumers: A cross-sectional study in Kampala city, Uganda. Nutr Health. 2020 Sep;26(3):187–95.
6. Zainal Arifen ZN, Haron H, Shahar S, Harun Z, Michael V, You YX, et al. Perceptions, barriers and enablers on salt reduction in the out-of-home sectors in Malaysia (MySaltOH) from the perspective of street food vendors, caterers and consumers. Public Health Nutr. 2024;27(1):e12.
7. WHO/PAHO Regional Expert Group for Cardiovascular Disease Prevention through Population-wide Dietary Salt Reduction Sub-group for Research and Surveillance. Protocol for Population Level Sodium Determination in 24-Hour Urine Samples. 2010.
8. Bartoshuk LM, Duffy VB, Green BG, Hoffman HJ, Ko CW, Lucchina LA, et al. Valid across-group comparisons with labeled scales: the gLMS versus magnitude matching. Physiology & Behavior. 2004 Aug;82(1):109–14.
9. Schutz HG, Cardello AV. A LABELED AFFECTIVE MAGNITUDE (LAM) SCALE FOR ASSESSING FOOD LIKING/DISLIKING [1]. Journal of Sensory Studies. 2001 Apr;16(2):117–59.
10. WHO/IOTF/IASO. The Asia-Pacific perspective: redefining obesity and its treatment. World Health Organization, International Obesity Task Force, International Association for the Study of Obesity; 2000.
11. Omron. Instruction Manual - Body Composition Monitor Model HBF-375 KaradaScan (TM) [Internet]. [cited 2023 Jul 25]. Available from: https://www.omronhealthcare-ap.com/Content/uploads/products/789b8222779742fe808151a86d9851e4.pdf
12. WHO. Waist Circumference and Waist–Hip Ratio: Report of a WHO Expert Consultation [Internet]. WHO Expert Consultation; 2011 [cited 2023 Jul 25]. Available from: https://apps.who.int/iris/bitstream/handle/10665/44583/9789241501491_eng.pdf

MALAYSIAN SOCIETY OF HYPERTENSION
(Persatuan Hipertensi Malaysia)
The Secretariat
A Menarini Spore Pte Ltd
B-18-2, Level 18, The Ascent Paradigm,
No.1, Jalan SS7/26A, Kelana Jaya,
47301 Petaling Jaya, Selangor Darul Ehsan, Malaysia
Tel: 03 – 79857000 | msh.secretariat.m@gmail.com
www.msh.my

2023 / 2025 Council Members

President
Dato' Sri Dr. Azhari Rosman

Vice-President
Prof. Dr. Nik Sherina Hanafi

Hon General Secretary
Dato' Wira Dr. Chandran

Hon Treasurer
Assoc. Prof. Dr. Ching Siew Mooi

Council Members
Prof. Datin Dr. Chia Yook Chin Dr. Chong Kuck Meng
Prof. Dr. Khoo Ee Ming
Dr. Navin Kumar A/L Devaraj
Dr. Beh Hooi Chin
Dr Iskandar Mirza

22nd July 2024

Dr Daniel Cheng Maong Hui
Department of Medical Sciences,
School of Medical & Life Sciences,
Sunway University

Dear Dr Daniel Cheng Maong Hui
RE: Grant Number Research MSH 2024/ 01- For Sunway University

I am pleased to inform you that this is the APPROVAL letter for your Research Grant Request for RM 20, 000 from the Malaysian Society of Hypertension.
[Grant Number Research MSH 2024/01]

THANK YOU

Yours Sincerely

Chandran

Dato Wira Dr Chandran
Hon. General Secretary
Malaysian Society of Hypertension

Cc Dato Sri Dr Azhari Rosman
President
Malaysian Society of Hypertension

Prof Ching Siew Mooi
Hon. Treasurer
Malaysian Society of Hypertension
